# A dyadic daily diary investigation of partner-schema structures on relational well-being and depressed mood

**DOI:** 10.1177/02654075251407064

**Published:** 2025-12-09

**Authors:** Gabriela C. M. Murphy, Fei Ying, Owen Hicks, Jessica A. Maxwell, David J. A. Dozois

**Affiliations:** 1 6221 Western University, Canada; 2 3710 McMaster University, Canada

**Keywords:** Daily diary, depression, relationship well-being, romantic relationships, schemas

## Abstract

Partner-schemas structures (i.e., the degree of interconnectedness in the beliefs one holds about their romantic partner) have been associated with relationship well-being and, to a lesser degree, depression, in cross-sectional research. However, little is known about how schema structures may impact couples in their daily lives. To address this gap, 260 couples were sampled at baseline to assess partner-schema structures, then surveyed for 14 days. Daily surveys captured relationship quality, relationship conflict severity, and relationship rumination; changes in relationship quality and relationship rumination on the day of a relational conflict; and depressed mood. Using multilevel modelling, guided by the Actor-Partner Interdependence Model, significant actor and partner effects emerged. Generally, the results suggest that an individual’s partner-schema structures are significantly associated with their own daily relationship quality, relationship rumination, increased relationship rumination on the day of a relational conflict, and depressed mood. Two partner effects also emerged, indicating that one’s own partner-schema structures may shape one’s partner’s relationship rumination on the day of a conflict, as well as their partner’s depressed mood. Exploratory analyses also suggest that the linkages between conflict severity and daily relationship quality, daily relationship rumination, and daily depressed mood may be moderated by partner-schema structures. These findings extend previous cross-sectional research to the daily dyadic context. Implications for future research are discussed.

Romantic relationships are associated with a host of positive outcomes. Higher relationship quality is positively correlated with benefits such as self-esteem, physical health, and life satisfaction, both cross-sectionally and longitudinally (Braithwaite & Holt-Lunstad, 2017). It is therefore perhaps unsurprising that researchers have also demonstrated significant associations between relationship distress and depression. For example, individuals with depression report higher incidences of interpersonal problems and distress (Schneider, 2014). Moreover, a recent machine-learning investigation of over 100 predictors of relationship quality across 43 longitudinal datasets found that depressive symptoms were among the top individual predictors of relationship quality (Joel et al., 2020). Indeed, a growing body of research demonstrates that depressive symptoms are a strong and consistent predictor, and outcome, of low relationship quality (e.g., Davila et al., 1997; Gustavson et al., 2012; Morgan et al., 2018) — a finding which has been replicated globally (see Whisman et al., 2021, for review). Given the impact romantic relationships have on individual well-being, understanding vulnerability factors that contribute to relationship distress and depression is critical. One key factor reliably linked to both depression and, more recently, relationship dysfunction, are schemas (Dozois, 2021).

## Schemas as cognitive vulnerabilities to depression and relationship well-being

Decades-worth of research has consistently implicated schemas, broadly defined as “the basic structural components of cognitive organization through which humans come to identify, interpret, categorize, and evaluate their experiences” (Schmidt et al., 1999, p. 129), as a key cognitive vulnerability associated with depression (Dozois & Beck, 2023). Schemas are largely stable across time (Dozois, 2007; Dozois & Dobson, 2001a; Seeds & Dozois, 2010; Wilde, 2023) and can be understood in terms of their content (positive versus negative) and structure (highly versus loosely organized or interconnected). Specifically, the schemas an individual holds about themself (i.e., self-schemas), and their organization (i.e., self-schema structures [SSS]) — often assessed using the Psychological Distance Scaling Task (PDST) — have been reliably implicated in depression, whereby individuals with depression consistently demonstrate highly interconnected negative SSS and loosely interconnected positive SSS (see Dozois & Beck, 2023, for review). Within this literature, schema structures are theorized to represent interconnected networks of beliefs or characteristics (e.g., I am kind, warm). Additionally, cognitive models often propose that highly interconnected, or well-consolidated, schema structures are more readily activated, which may result in a spreading of congruent information that impacts individuals’ thoughts and behaviors (e.g., Dozois & Beck, 2023).

Despite clear associations with depression, SSS do not appear to be as strongly associated with relational outcomes such as commitment and satisfaction (Wilde & Dozois, 2018). Given the relations between depression and relationship distress, the lack of strong associations between schema structures and relationship quality is puzzling. However, to-date, research on schemas and depression has largely focused on *self-*schema structures, whereas the role of schema structures about romantic partners has received relatively little attention (Chatav & Whisman, 2009; Wilde & Dozois, 2018). Partner-schemas — defined as “cognitive generalizations, derived from past experience, that organize and guide the processing of partner-related information” (Whisman & Delinsky, 2002, p. 619) — can also be measured in terms of their content and organization. Partner-schema structures (PSS) have been similarly assessed with the PDST (partner version). Upon completion of this task, an individual receives two PSS scores: a positive partner-schema structure score and a negative partner-schema structure score, with each score representing the distance, or degree of interconnectedness, of the positive and negative schema structures they hold for their partner. To illustrate, consider the hypothetical couple, Jordan and Mona. If Mona has little distance among the adjectives that form their negative partner-schema structure of Jordan on the PDST (e.g., beliefs that Jordan is lazy, immature, and hot-tempered), this arguably represents more tightly interconnected (or highly organized) negative cognitive generalizations about Jordan.

Preliminary work suggests that loosely interconnected positive PSS and highly interconnected negative PSS are correlated with depressive symptoms (though to a smaller degree than are SSS; Wilde & Dozois, 2018) and importantly, predictive of several relational measures such as relationship satisfaction, commitment, and adjustment, over and above SSS (Murphy et al., 2025; Wilde & Dozois, 2018). Moreover, previous work suggests that partner-schemas may be predictive of distress-maintaining attributions for a partner’s behavior and marital satisfaction (Chatav & Whisman, 2009; Whisman & Delinsky, 2002).

To integrate the various findings on depression, relationship functioning, and partner-schemas, Wilde and Dozois (2019) proposed the Dyadic Partner-Schema Model (DPSM). This novel theoretical framework proposes various processes through which self- and partner-schema structures may shape an individual’s perceptions about themself and their partner, and how these perceptions may lead to maladaptive interpersonal behaviors (e.g., through biased in-vivo cognitions or attributions about one’s partner, see Wilde & Dozois, 2019, for details). To illustrate, returning to Jordan and Mona, if Mona calls Jordan to find out why he did not pick up the kids from school, this may activate Mona’s belief that Jordan is lazy, which may quickly spread across her tightly interconnected negative partner-schema structure, and activate other beliefs (e.g., Jordan is immature). The DPSM theorizes that in this interpersonal situation, Mona’s schema for Jordan serves as a filter for incoming information (e.g., Jordan’s response), biases Mona’s cognitions (e.g., not believing Jordan was held up at work; blaming Jordan), and impacts her behaviors (e.g., engaging in interpersonal conflict).

Since the DPSM’s conceptualization, longitudinal work spanning six months suggests that PSS remain relatively stable (Wilde, 2023). Although this understanding of the stability of partner-schemas across time is invaluable, it provides little information on how schematic structures impact dyadic well-being in everyday life. Indeed, previous research has called for an investigation of schema structures on daily stressors, mood, and interpersonal processes (e.g., Seeds & Dozois, 2010; Wilde, 2023), and noted the potential utility for such investigations to be conducted within dyadic samples (e.g., Wilde & Dozois, 2019).

## The importance of daily diary methodology

Daily diary methodology may offer one of the best approximations of the processes individuals experience in their everyday lives (Bolger et al., 2003; Galovan et al., 2023), thus providing a useful tool to examine the role of partner-schemas in shaping daily relationship experiences. By inquiring about individuals’ experiences (e.g., feelings about their relationships, how often they ruminate about their relationship, and the severity of relational conflicts) each day, daily surveys provide important insight into the ecological validity (Lischetzke, 2014) of PSS and their role within romantic relationships.

### Daily relationship well-being

Relationship quality is among the most widely used constructs of relationship well-being (Fowers et al., 2016). However, participants’ assessments of their relationship quality are prone to bias (e.g., negative retrospective bias; Peetz et al., 2022). Considering this bias, assessing relationship quality at multiple time points using daily surveys, for example, is likely to offer a more accurate assessment than measuring relationship quality at one time point.

Other relevant facets of relationship well-being may also benefit from daily assessments. Relationship rumination, for instance, is a recently developed index of relationship well-being, and may be particularly relevant to understanding cognitive vulnerabilities, such as schema structures, because ruminating about one’s relationship is likely to involve the underlying networks that hold partner-related representations (i.e., PSS, Machado, 2024). Rumination more broadly can be defined as “repetitive, prolonged, and recurrent negative thinking about one’s self, feelings, personal concerns and upsetting experiences” (Watkins & Roberts, 2020, p. 1). Akin to rumination being associated with depression (Watkins & Roberts, 2020), ruminating specifically about ones’ relationship may be associated with relationship adjustment (see Senkans et al., 2016, for a review). Indeed, preliminary research indicates that relationship rumination (e.g., worrying about what is wrong with one’s partner or relationship) is negatively associated with relationship satisfaction and commitment (Elphinston et al., 2013; Machado, 2024). Moreover, past research suggests that rumination is prone to variation across time (Hankin, 2008) and can be considered state-like; fluctuating in response to various daily experiences such as interpersonal offenses (Kirkegaard, 2006; Machado, 2024; Wade et al., 2008). As such, the measurement of relationship rumination may benefit from daily assessments.

Relationship conflict provides another useful gauge of relationship well-being. Although conflict may be helpful and, at times, necessary within romantic relationships (Laursen & Hafen, 2010), relational conflicts often correlate positively with depressive symptoms (Mackinnon et al., 2012), and negatively with relationship quality (Kluwer & Johnson, 2007). Conflict severity may be especially important to consider, perhaps more so than the number of conflict occurrences, which cannot distinguish between mild and severe conflicts (Kluwer & Johnson, 2007). Methodologically, it is imperative to capture relational conflicts that arise spontaneously in couples’ daily lives (Sheng et al., 2022), because conflict generated in a lab setting (e.g., through prompts or manipulation) often underestimates, and does not generalize to, couples’ conflict experiences outside of the lab (Laurenceau & Bolger, 2005). Consequently, daily diary methodology affords more generalizable estimates of the influence of partner-schemas on everyday conflict experiences. Furthermore, daily assessments allow for analyses on whether PSS moderate associations between conflict experiences and relationship quality (Kluwer & Johnson, 2007), as well as between conflict experiences and depressive mood (Mackinnon et al., 2012) in daily life.

### Changes in daily relationship well-being when conflicts occur

In addition to the increased sensitivity gained by measuring relationship well-being each day (e.g., reducing biased recall), daily diary methodology allows for critically needed lagged analyses (Li et al., 2018) between relational experiences and subsequent relationship well-being. Indeed, previous research utilizing daily diaries found that perceived relationship quality may fluctuate with, and in response to, experiences in couple’s lives (e.g., conflict, sexual experiences; Campbell et al., 2010). Importantly, such fluctuations in relationship perceptions may offer key insight into relational health, with previous work suggesting that variability in relationship perceptions may be “uniquely harmful” to relationship health (Cooper et al., 2018, p. 349). Assessing relationship well-being with daily diaries allows for evaluations of contextual factors (e.g., conflict experiences) that predict fluctuations in relationship well-being, while controlling for relationship well-being the day prior.

### Depressive mood

Daily diary methods may similarly offer more precise assessments of depressed mood. Horwitz et al. (2023), for instance, found that using measures that ask participants to recall depressive symptoms across longer time frames (e.g., two weeks), results in systemic recall bias that overemphasizes the peak (i.e., worst) and end (i.e., current) mood states at the time of measurement. In studies that measure depressive symptoms more frequently (e.g., daily), depressive symptoms change in response to everyday experiences among both individuals with and without depression (e.g., Pâquet et al., 2018; Peeters et al., 2003).

## The need for dyadic data

Most studies examining links between relationship well-being, depression, and associated constructs (e.g., cognitive vulnerabilities) utilize data from only one member of the couple (Gilmour et al., 2022). However, a more detailed and nuanced understanding of these constructs may be obtained by collecting data from both members of the dyad (Gilmour et al., 2022; Pâquet et al., 2018). Indeed, as theorized within the DPSM, PSS impact one’s cognitions (e.g., thinking that a partner is to blame) and subsequent behaviors (e.g., hostility) toward a partner, which may, in turn, shape both partners' relationship well-being and depressive symptoms (Wilde & Dozois, 2019). Although assessments of such in-vivo cognitions (e.g., blame attributions) are beyond the scope of the present study, dyadic data offer the ability to begin to uncover whether PSS are associated not only with one’s own, but one’s *partner’s*, daily experiences.

In summary, romantic relationship distress and depression are highly interrelated, and previous work suggests that schemas are an important vulnerability factor underlying relationship quality and depressive symptoms. Nevertheless, research examining the role of schema structures in relational and mental well-being has largely been limited to cross-sectional methodologies in non-dyadic samples. To address these gaps, the present study represents the first in-depth examination of PSS on daily relationship well-being, changes in relationship quality and relationship rumination on the day of a conflict, and daily depressed mood in a dyadic sample.

## Present study

The primary aim of this study was to investigate the associations between baseline PSS and daily relationship well-being (operationalized as daily reports of relationship quality, relationship rumination, conflict severity), as well as between PSS and changes in daily relationship quality and relationship rumination on the day of a relational conflict.

In preregistered hypotheses, we predicted that more loosely organized positive PSS and more highly organized negative PSS at baseline would be associated with lower daily relationship quality, as well as greater daily relationship rumination and conflict severity. We also predicted that more loosely organized positive PSS and more highly organized negative PSS would be associated with lower relationship quality and, in a separate model, greater relationship rumination on the day of a conflict occurrence, after controlling for relationship quality and relationship rumination the day prior.^1^

Additionally, despite previous work suggesting that SSS may be a better predictor of depressive symptoms than PSS (e.g., Wilde & Dozois, 2018), to our knowledge, no work has examined the role of PSS within the broader category of depressed mood (i.e., state-level feelings of sadness or “feeling blue”), nor have such associations been explored within dyadic or daily contexts. Thus, although not preregistered, we also conducted exploratory analyses investigating whether PSS were associated with daily depressed mood.

Within all models, we examined whether there were any partner effects of PSS. Although partner effects within relationships and depression research have been found (e.g., Aguilar-Raab et al., 2022; Beach et al., 2003; Gilmour et al., 2022), they are often much smaller than actor effects and less likely to emerge in dyadic models (Eastwick et al., 2023). Considering that little is known regarding PSS within the daily dyadic context, these analyses, although preregistered, were exploratory in nature.

As part of our preregistered analytic plan, we also examined a set of exploratory models testing whether PSS moderate the associations of daily conflict severity on relationship quality and depressed mood. Although not central to the present study, these supplementary models may offer preliminary insight into potential contexts through which schema structures shape relational and emotional experiences in everyday life (see Supplement). Finally, across all models, we tested whether any significant effects persisted when controlling for relationship length and SSS (see Supplement). Unless otherwise noted, all hypotheses, measures, and data analytic plans were preregistered on the Open Science Framework prior to beginning data collection, where anonymized data and syntax have also been uploaded (https://osf.io/prav2).^2^

## Method

### Participants

Couples were recruited through advertisements in various locations around London, Ontario (e.g., libraries, medical offices), and through social media (e.g., Facebook). A total of 298 couples enrolled, all of whom elected to complete the daily diary surveys after completing the baseline survey. Five couples were unable to finish the baseline survey for a variety of reasons (e.g., not fluent in English, had to leave early), and were consequently not invited to participate in daily diary assessments. After data collection was complete, nine couples were removed because one or both members of the dyad completed one or fewer daily diary entries, and 24 couples were removed because one or both members were missing either positive or negative PSS (see Supplement for details). The final sample consisted of 520 participants, or 260 dyads (265 individuals identified as cisgender women, 233 as cisgender men, 16 as non-binary, one as a transgender woman, one as a transgender man, one as genderqueer, one as gender non-conforming, one as gender-fluid, and one as Two-Spirit). Participants ranged in age from 18 to 70 (*M* = 27.71, *Median* = 24.00, *SD* = 9.82), with relationship length varying between 3 months and 43.33 years (*M* = 4.63 years, *SD* = 6.45). Participants were also highly educated (*M* = 15.46 years of education, *SD* = 2.88). Participants were diverse with respect to their ethnic background (50.8% of participants identified as White, 13.8% as East Asian, 12.8% as South Asian, 7.4% as Multi-Ethnic, 5.4% as Middle Eastern, 4.7% as Latin American, 1.7% as South East Asian, 1.9% as Black, 0.6% as Caribbean, 0.4% as Indigenous, 0.4% as “Other” and 0.2% as “Unknown”), and sexual identity (67.6% identified as heterosexual, 12.6 % as bisexual, 4.7% as lesbian, 4.3% as asexual, 3.5% as pansexual, 3.1% as queer, 1.7% as gay, 1.9% as uncertain or questioning, 0.4% selected ‘other’ [0.2% wrote straight demisexual, 0.2% wrote polyamorous], and 0.2% chose not to specify). Most participants (61.9%) were seriously dating one person, 27.3% were married or common-law, 6.3% were engaged, 2.3% were casually dating one person, 0.8% were seriously dating more than one person, 0.8% reported “other”, and 0.6% were casually dating more than one person. Finally, 220 couples were composed of individuals who identified as a cisgender woman and a cisgender man, five couples were composed of two cisgender men, 16 couples of two cisgender women, and 19 couples contained at least one gender diverse member.

### Measures

#### Baseline measures

##### Partner-schema structures

Schema structures were assessed using the Psychological Distancing Scaling Task (PDST) – partner version (Dozois, 2002, 2007; Wilde & Dozois, 2018). Modified from the self-version of the PDST, which has been well-validated across both clinical and community samples (e.g., Diehl et al., 2017; Dozois & Dobson, 2001b), the partner-version demonstrates convergent, incremental, and divergent (from attachment orientations) validity in community samples (Murphy et al., 2025). In this task, participants were presented with a small rectangular grid on a computer screen, with the x-axis showing the statements “*Not at all like my partner*” on the left, and “*Very much like my partner*” on the right. The perpendicular y-axis shows the statements “*Very positive*” at the top, and “*Very negative*” at the bottom. One at a time, and in random order, 60 adjectives (30 positive, 30 negative) appeared on the screen and participants were asked to place a dot on the grid in a location that they felt accurately described their partner (see Supplement for details).

Positive and negative partner-schema structures were calculated using the x/y coordinates of each adjective. Interstimulus distances scores (ISD) were computed using an idiographic formula, resulting in two schema structure values for each participant (i.e., positive partner and negative partner). Higher ISD scores (i.e., greater distance among adjectives) are thought to reflect less consolidation, or interconnectedness, of information, whereas lower ISD scores (i.e., less distance among adjectives) indicate greater consolidation, or interconnectedness, of information (Dozois, 2021; Dozois & Frewen, 2006). Thus, a *high* positive partner ISD score denotes *greater* distance among positive adjectives, and is therefore thought to represent a *less* consolidated, or interconnected, positive PSS. Meanwhile, a *low* positive partner ISD score would denote *less* distance between positive adjectives, and therefore is assumed to represent a *more* consolidated, or interconnected, positive PSS. The same remains true for negative PSS, calculated as the distance between negative adjectives in the PDST. For a detailed description of the PDST and its formulaic development, see Dozois and Dobson (2001b) or Seeds and Dozois (2010).

As expected, based on previous work (e.g., Seeds & Dozois, 2010; Wilde, 2023), ISD scores for PSS were highly skewed. As with all studies utilizing the PDST (e.g., Diehl et al., 2017; Wilde & Dozois, 2018), and as recommended by the developers of the PDST (Dozois & Dobson, 2001a, 2001b), all scores for each participant were logarithmically transformed with a base-10 transformation. These tasks resulted in two PSS scores for each participant: a positive PSS score (*M* = 0.93, *SD* = 0.18), and a negative PSS score (*M* = 1.56, *SD* = 0.35). Finally, as has occurred in previous work utilizing the PDST (e.g., Wilde, 2023; Wilde & Dozois, 2018), a small number of participants did not endorse any of the positive and/or negative adjectives as partner-referent. In such instances, the participant did not receive an ISD score for that particular valence and was, therefore, excluded from analyses (see Supplement for details).

#### Daily diary measures

##### Relationship well-being indices

Relationship quality was measured by averaging responses to the most face-valid item (e.g., “How satisfied are you with your relationship today?”) from each of the six subscales (i.e., satisfaction, intimacy, passion, commitment, love, and trust) of the Perceived Relationship Quality Component Inventory (PRQC; Fletcher et al., 2000; *Rc =* .75 *M* = 5.89, *SD* = 1.03), as recommended by the scale developers. These items were assessed on a seven-point scale, ranging from 1 = *strongly disagree* to 7 = *strongly agree*.

Relationship rumination was measured using the most face valid item that displayed the highest loading from the Partner Rumination Scale (Machado, 2024). This item was then adapted for the daily diary format. Specifically, participants were asked to rate, on a scale ranging from 1 = *does not describe me well* to 7 = *describes me very well*, the following statement, “I spent a lot of time thinking about the things that are wrong with my relationship today” (*M* = 1.70, *SD* = 1.32).

Relationship conflict was measured by asking participants, “Did a conflict or disagreement of any kind occur with your partner today?” Participants could select *No* or *Yes*. If participants indicated that a conflict occurred, conflict severity was assessed with the following question, “How serious/damaging was the conflict/disagreement with your partner today?”, assessed on a scale ranging from 1 = *not at all* to 7 = *extremely* (*M* = 2.57, *SD* = 1.52). Because conflict may influence relationships even if perceived by only one couple member, couples were considered to have a conflict if one or both partners reported a conflict occurrence, as has been done in previous research (e.g., Maxwell & Meltzer, 2020).

##### Depressed mood

Finally, daily depressed mood was assessed on a scale ranging from 1 = *not at all* to 7 = *extremely* (*M* = 2.20, *SD* = 1.54) by asking participants, “How down/depressed/or blue did you feel today?” Previous work has highlighted that short and even single-item questionnaires inquiring about depression offer significant utility in detecting depression and depressive mood in both clinical and non-clinical samples (Dozois et al., 2020; Mitchell, 2008).

### Procedure

This study was approved by Western University (Ethics Approval #: 119730). Data collection began in August 2022 and was completed in April 2024.^3^ To qualify for this study, both members of the dyad were required to be in a relationship for three months or longer, at least 18 years old, fluent in English, and without a history of intimate partner violence. Participants’ eligibility was determined via an initial pre-screening survey.

Eligible couples completed consent forms, demographic, and baseline surveys in-lab, each in separate, private testing rooms. Beginning on the day following their in-lab session, participants were sent an individualized link to a two-minute daily survey every evening for 14 consecutive days. Participants were able to complete each survey between 5:00 p.m. that day and 10:00 a.m. the following day; however, they were encouraged to complete the survey in the evening. Participants earned $30 CAD for the baseline survey, as well as $2 CAD and one ballot entry for a chance to win one of seven, $100 CAD Amazon gift cards for each diary entry completed. Following the two-week daily diary period, participants were sent a debriefing form.

### Data analytic approach

**Figure 1.**
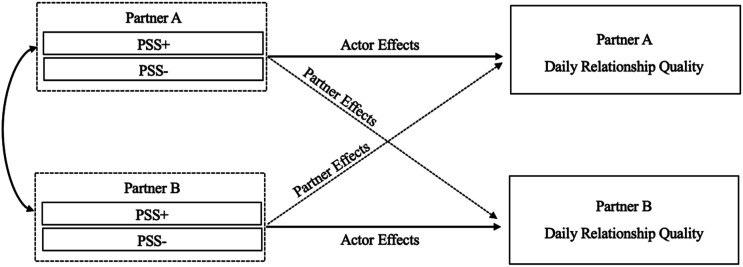
Theoretical APIM model. *Note.* PSS+ = positive partner-schema structure; PSS− = negative partner-schema structure.

Analyses were performed using IBM SPSS Statistics, Version 29. The main models were analyzed with Multilevel Modeling (MLM), guided by the Actor-Partner Interdependence Model (APIM; Kenny et al., 2006). Specifically, we tested APIM two-level cross-classified models,^4^ with persons nested within dyads, and persons and days crossed to account for the fact that both partners completed surveys on the same days (Horne et al., 2022; Kenny et al., 2006). Consistent with previous work (e.g., Wilde, 2023; Wilde & Dozois, 2018), positive and negative PSS were simultaneously entered in each model. Separate models were run for each outcome variable, resulting in six main models (see Figure 1 for a sample model). To examine the effect of PSS predicting residual changes in (a) relationship quality and (b) rumination on the day of a conflict, we controlled for relationship quality and rumination the day before a conflict occurrence by creating lagged variables. All continuous baseline predictors and covariates (i.e., PSS, lagged relationship quality and rumination, and relationship length) were grand-mean centered. See Supplement for details on effect size calculations.

As this sample included both mixed- and same-gender couples, and there are no theoretical reasons to suspect that results would differ between mixed- and same-gender couples, all dyads were considered indistinguishable. Each model simultaneously examined both actor effects (e.g., the association between one’s own PSS and own daily relationship well-being), and partner effects (e.g., the association between one’s partner’s PSS and one’s own daily relationship well-being). All models were tested with a random intercept for each couple, with no other random effects.

## Results

### Descriptive analyses

Across the 520 participants, a total of 6398 diary entries were completed (out of a total of 7280 possible diary entries), resulting in a completion rate of 87.88% (*M* = 12.30 out of 14 diaries). A total of 533 conflicts were captured across the 260 dyads, with an average of 2.05 conflicts reported per couple across the 14-day study period (*SD* = 1.68; *Range* = 0–12 days with conflicts). See Supplement for bivariate correlations among key variables (Table S1).

### Main model results

Main model results are provided in Tables 1–4. Within these tables, PSS + describes positive partner-schema structure interstimulus distance; PSS- describes negative partner-schema structure interstimulus distance.

**Table 1.** PSS predicting actors’ daily relationship quality and relationship rumination.

	Actor daily relationship quality						Actor daily relationship rumination					
Effects	*b*	*SE*	*t(df)*	*p*	95% CI	Effect size *r*	*b*	*SE*	*t(df)*	*p*	95% CI	Effect size *r*
Actor												
PSS+	−0.99	0.18	−5.38 (273.22)	<.001	[−1.35, −0.63]	0.31	0.60	0.19	3.17 (298.98)	.002	[0.23, 0.97]	0.18
PSS−	0.04	0.10	0.40 (272.91)	.691	[−0.15, 0.23]	0.02	−0.48	0.10	−4.73 (297.56)	<.001	[−0.67, −0.28]	0.26
Partner												
PSS+	−0.33	0.18	−1.77 (274.27)	.079	[−0.69, 0.04]	0.11	0.15	0.19	0.80 (301.68)	.424	[−0.22, 0.52]	0.05
PSS−	0.06	0.10	0.57 (272.77)	.567	[−0.14, 0.25]	0.03	−0.17	0.10	−1.66 (297.26)	.098	[−0.36, 0.03]	0.10

**Table 2.** PSS predicting actors’ daily relationship quality on conflict days.

	Actor daily relationship quality on the day of a conflict					
Effects	*b*	*SE*	*t(df)*	*p*	95% CI	Effect size *r*
Actor						
PSS+	−0.23	0.2	−1.13 (246.13)	.259	[−0.62, 0.17]	0.07
PSS−	0.12	0.11	1.12 (209.70)	.265	[−0.09, 0.33]	0.08
Partner						
PSS+	−0.14	0.20	−0.67 (256.70)	.501	[−0.54, 0.26]	0.04
PSS−	0.07	0.11	0.68 (211.86)	.500	[−0.14, 0.28]	0.05
Actor lagged RQ	0.64	0.03	18.73 (590.08)	<.001	[0.57, 0.71]	0.61

*Note.* Actor Lagged RQ = actor’s relationship quality the day prior.

**Table 3.** PSS predicting actors’ daily relationship rumination on conflict days.

	Actor daily relationship rumination on the day of a conflict					
Effects	*b*	*SE*	*t(df)*	*p*	95% CI	Effect size *r*
Actor						
PSS+	0.68	0.36	1.88 (286.41)	.061	[−0.03, 1.38]	0.11
PSS−	−0.39	0.19	−2.02 (263.23)	.044	[−0.78, −0.01]	0.12
Partner						
PSS+	0.13	0.37	0.35 (308.2)	.725	[−0.6, 0.86]	0.02
PSS−	−0.42	0.19	−2.17 (263.50)	.031	[−0.81, −0.04]	0.13
Actor lagged RR	0.39	0.04	10.27 (796.56)	<.001	[0.32, 0.47]	0.34

*Note.* Actor Lagged RR = actor’s relationship rumination the day prior.

**Table 4.** PSS predicting actors’ daily depressed mood.

	Actor daily depressed mood					
Effects	*b*	*SE*	*t(df)*	*p*	95% CI	Effect size *r*
Actor						
PSS+	0.79	0.20	3.95 (324.92)	<.001	[0.40, 1.19]	0.21
PSS−	−0.34	0.11	−3.15 (322.00)	.002	[−0.55, −0.13]	0.17
Partner						
PSS+	0.55	0.20	2.73 (328.56)	.007	[0.15, 0.95]	0.15
PSS−	−0.01	0.11	−0.12 (321.35)	.904	[−0.22, 0.20]	0.01

#### Daily relationship quality

In line with our hypothesis, actors’ positive PSS at baseline was significantly associated with actors’ daily relationship quality, such that more interconnected positive PSS (i.e., a lower PSS+ ISD) was associated with higher relationship quality. However, contrary to our hypothesis, actors’ negative PSS was not significantly associated with actors’ daily relationship quality. Partners’ PSS did not demonstrate any significant effects on actors’ daily relationship quality (see Table 1).

#### Daily relationship rumination

As hypothesized, actors’ less interconnected positive PSS (i.e., a lower PSS+ ISD) and more interconnected negative PSS (i.e., a higher PSS- ISD) at baseline were associated with greater actor daily relationship rumination (see Table 1). No significant partner effects emerged between partner PSS and actors’ daily relationship rumination.

#### Relationship quality and relationship rumination on the day of a conflict occurrence

Contrary to predictions, actors’ and partners’ positive and negative PSS were not significantly associated with relationship quality on the day that a conflict occurred, after controlling for relationship quality the day prior (see Table 2). Also contrary to predictions, actor and partner positive PSS were not associated with relationship rumination on the day of a conflict, after controlling for relationship rumination the day prior. However, more interconnected actor and partner negative PSS (i.e., lower ISD) were associated with greater actor daily relationship rumination on the day of a conflict, after controlling for the actor’s relationship rumination on the day prior to a conflict (see Table 3).

#### Daily conflict severity

We hypothesized that actors’ more loosely organized positive PSS and more highly organized negative PSS at baseline would be associated with greater conflict severity. Contrary to predictions, no significant actor or partner associations emerged (see Supplemental Table S2).

#### Daily depressed mood

In an exploratory model examining PSS and daily depressed mood, actors’ loosely interconnected positive PSS and highly interconnected negative PSS at baseline were associated with higher actor reports of daily depressed mood. Additionally, partners’ highly interconnected positive PSS was significantly associated with actors’ lower daily depressed mood (see Table 4).

### Supplementary analyses

#### PSS as moderator

In exploratory supplementary analyses, we examined whether PSS moderated the associations between conflict severity and that day’s relationship quality, between conflict severity and that day’s daily relationship rumination, and between conflict severity and that day’s depressed mood. For conflict severity, we parsed apart within-person variance (within-person centered to indicate whether the conflict severity a participant reported on a given day was higher or lower than their own average level of conflict severity) and between-person variance (between-person centered to indicate whether a person reported higher conflict severity across the diary period relative to other participants). Eight significant interactions emerged. At the within-person level of conflict severity, simple slope analyses (i.e., ±1 *SD* of positive and negative PSS ISD) revealed that the negative association between actor’s and partner’s daily conflict severity and actor’s daily relationship quality was non-significant for actors and partners with a more tightly interconnected positive PSS (i.e., −1 *SD* positive PSS ISD; see Figures S1A and S1B). This contrasts with the pattern of results for daily rumination, in which the positive association between partners’ daily conflict severity and actors’ daily relationship rumination was significant only for actors with a tightly interconnected positive PSS (i.e., −1 *SD* positive PSS ISD; see Figure S1C). When considering the between-person level of actors’ conflict severity, the negative association between average conflict severity and relationship quality was amplified when partners held a more tightly interconnected positive PSS (see Figure S2A), when actors held a more tightly interconnected negative PSS (see Figure S2B), and when partners held a more loosely interconnected negative PSS (see Figure S2C). With regard to depressed mood at the between-person level of conflict severity, the positive association between actors’ average conflict severity and actors’ daily depressed mood was amplified when partners held a more loosely interconnected positive PSS (see Figure S2D), as well as a more loosely interconnected negative PSS (see Figure S2E). Full results for these supplementary analyses can be found in the Excel Online Supplement.

#### Generalizability of findings

All significant effects described in the results section remained when controlling for relationship length, apart from actor (*p =* .078) and partner negative PSS (*p =* .059) predicting relationship rumination on the day of a conflict (see Supplement). Although previous work (Murphy et al., 2025) indicates that SSS demonstrate discriminate validity from PSS, as outlined in our preregistration, we also controlled for SSS in all main models. Most significant findings remained; however, the associations between actor negative PSS predicting relationship rumination on the day of a conflict (*p =* .060) and actor negative PSS predicting daily depressed (*p =* .070) mood were no longer significant at *p* < .05 (see Supplement). Moreover, positive actor PSS predicting daily depressed mood was no longer significant. By and large our pattern of findings was robust to relationship length and SSS.

## Discussion

In a sample of community dyads, we examined the role of baseline PSS on daily relationship well-being, changes in relationship quality and relationship rumination on the day of a relational conflict, and depressed mood. Consistent with our hypotheses and previous research outside of the daily context (e.g., Wilde & Dozois, 2018), positive PSS significantly predicted daily relationship quality. Extending previous work, PSS also predicted daily relationship rumination, relationship rumination on the day of a conflict experience, and daily depressed mood. In contrast, PSS did not significantly predict conflict severity, nor relationship quality on the day of a conflict experience.

### Daily relationship quality

As hypothesized, individuals with more highly interconnected positive beliefs about their partner were more likely to report higher daily relationship quality, and individuals with more loosely interconnected positive schemas about their partner were more likely to report lower daily relationship quality. This finding aligns with and extends previous cross-sectional work linking PSS and indices of relationship well-being by examining these associations on a daily level (e.g., Wilde & Dozois, 2018).

Conversely, negative PSS were not associated with daily relationship quality. This is puzzling, as previous research indicates that both positive and negative partner-schemas are associated with various indices of relationship functioning in non-dyadic, cross-sectional samples (e.g., Murphy et al., 2025; Whisman & Delinsky, 2002; Wilde & Dozois, 2018). These discrepant findings may stem from the generally high relationship quality ratings within the present sample (Park et al., 2021). It is possible that an insufficient number of individuals reported both highly interconnected negative PSS and low relationship quality to capture this association. Finally, no significant partner effects on daily relationship quality emerged. This may be a result of concerns raised by previous scholars that actor and partner reports of relationship quality often trend quite similarly (Joel et al., 2020) — a notion that aligns with the actor-partner correlations and findings within the present study. Ultimately, these findings partially concur with previous work (e.g., Wilde & Dozois, 2018) and affirm the importance of positive PSS in shaping individuals’ daily relationship quality.

### Daily relationship rumination

As expected, actors’ loosely interconnected positive PSS and highly interconnected negative PSS were associated with their higher daily relationship rumination. Although no partner effects emerged, the significant findings are particularly interesting as they suggest that PSS are associated with individuals’ daily negative thoughts about their relationship. In contrast to the vast literature examining self-focused rumination within the context of depression (Watkins & Roberts, 2020), a paucity of research has examined interpersonal and other forms of ruminative thought (Machado, 2024). Despite preliminary research that relationship rumination is a unique construct associated with a host of well-being outcomes (e.g., relationship quality; Machado, 2024), the current study was, to our knowledge, the first to examine daily relationship rumination, and to examine the association of schema structures on this unique facet of rumination.

### Relationship quality and relationship rumination on the day of a conflict occurrence

In models investigating relationship quality and rumination on conflict days, only actor and partner negative PSS predicted actor relationship rumination, after controlling for relationship rumination from the previous day. In other words, individuals with more highly interconnected negative PSS, and whose partners held more interconnected negative schema structures about them, were more likely to worry about what was wrong in their relationship on the day of a conflict occurrence. This finding supports the notion that relationship rumination may be considered state-like, rather than trait-like, and may fluctuate in response to daily interpersonal events (akin to general rumination e.g., Kirkegaard, 2006; Wade et al., 2008). Nevertheless, individuals’ relationship quality on the day of a conflict was statistically nonsignificant when controlling for relationship quality the day prior. Considering the low conflict severity scores, it is possible that the daily relational conflicts in the present sample may have been severe enough to result in individuals with highly interconnected negative PSS worrying more about their relationship, but not severe enough to impact their overall feelings about their relationship that day (i.e., daily relationship quality). Nevertheless, it is important to acknowledge that, given the relatively low number of days in which a conflict was reported by one or both members of the dyad (i.e., 14.6% of all possible diary entries were coded as dyadic conflict days), these models were less powered than other non-conflict-related models.

### Conflict severity

Contrary to hypotheses, actor and partner PSS were not associated with conflict severity. However, 59.1% of conflict severity reports fell below a 3 (out of 7) in the severity ratings. This smaller proportion of severe conflicts captured within the present sample across 14 days may at least partially account for these null findings. Other methods to assess relationship conflict (e.g., data of how couples engage in conflict) may be more appropriate for assessing conflict, particularly within healthy dyads (Busby & Holman, 2009).

Although PSS did not significantly predict conflict severity, preregistered supplementary analyses revealed that at times, actor and partner PSS moderated associations between conflict severity and actors’ relationship quality, relationship rumination,^5^ and actors’ depressed mood. At the within-person level, tightly interconnected positive PSS buffered the negative association between daily conflict severity and daily relationship quality. Specifically, when actors or partners held a more consolidated positive schema about their partner, actors were less likely to report lower daily relationship quality on days when conflict was more severe than usual compared to individuals who held a loosely interconnected positive PSS. This finding suggests a potential protective effect of positive partner schemas when couples engage in conflict that is more severe than their average. Individuals who hold tightly interconnected positive beliefs about their romantic partner, for example, may be less likely to process negative behaviors or assume negative intention behind their partner’s behaviors during a conflict (Dozois & Beck, 2023) and may demonstrate more constructive responses (Baldwin, 1992), which could reduce the impact of conflicts on perceptions of daily relationship quality. Meanwhile, individuals with a loosely interconnected positive PSS may be less likely to offer this same support or interpret conflicts in reparative ways, leaving them more vulnerable to lower daily relationship quality.

Compared to individuals with a more loosely interconnected positive PSS, however, actors with a more tightly interconnected positive PSS showed greater reactivity (i.e., a steeper, significant slope) in relationship rumination on days when their partners reported more conflict severity than typical. In contrast, individuals with a loosely interconnected positive PSS showed no significant change in relationship rumination as conflict severity rose (i.e., flat, non-significant slope). As reflected in Figure S1C, particularly when partners reported low severity conflict that day, those with looser positive PSS showed greater rumination than those with tighter positive PSS. Put another way, those with tightly interconnected positive PSS may differentiate their rumination based on conflict severity, whereas the rumination of those with loosely connected positive PSS was higher overall, and not contingent on partner reports of conflict severity that day. Together with the within-person results for relationship quality, this may suggest that a tightly interconnected positive PSS, compared to a more loosely interconnected positive PSS, may help buffer daily relationship quality across levels of within-person conflict severity; however, these tightly interconnected positive PSS may only buffer against lower relationship rumination when partners report less severe conflicts than usual.

Conversely, at the between-person level of conflict severity, interactions yielded mixed results. Partners’ tightly interconnected positive PSS partially buffered actors’ daily depressed mood among actors reporting more severe conflicts across the diary period yet were associated with greater declines in daily relationship quality. With regard to negative PSS, actors’ tightly interconnected negative PSS amplified the negative association between between-person conflict severity and daily relationship quality, whereas partners’ loosely interconnected negative PSS predicted steeper declines in daily relationship quality among actors reporting high average conflict severity. In contrast, partners’ tightly interconnected negative PSS partially buffered the negative association between actors’ average conflict severity and daily depressed mood. No significant associations predicting relationship rumination emerged. Together, the significant findings may reflect a double-edged nature of tightly held schemas: although they may stabilize emotional outcomes such as depressed mood, they may also amplify declines in relationship quality if rigidly upheld in the face of more chronic relational conflict, which could result in repeated expectancy violation (Burgoon, 2015). More broadly, these findings also underscore how PSS may be differentially associated with relational versus personal outcomes, aligning with previous work (Murphy et al., 2025). Given the mixed pattern of exploratory between-person effects however, these findings require replication before drawing strong conclusions.

In conjunction with the non-significant findings for PSS predicting conflict severity and changes in relationship quality on the day of a conflict, these supplementary analyses suggest that PSS may moderate the association between conflict severity on relationship quality and rumination in nuanced ways. Nevertheless, conflict severity, relationship quality, and rumination were reported concurrently, and thus temporality cannot be assumed. Moreover, the inconsistency of effects across actors and partners, outcomes, and level of analysis (i.e., within versus between) highlights the complexity of these dyadic processes and underscores the need to interpret these findings with extreme caution.

### Daily depressed mood

Considering the mounting evidence linking depression and romantic relationship distress (Whisman et al., 2021), we also explored the role of PSS on daily depressed mood. Although no a priori hypotheses were generated, both actors’ positive and negative PSS were associated with actors’ daily depressed mood. Moreover, a significant partner effect emerged whereby partners’ positive PSS significantly predicted actors’ daily depressed mood. These results extend previous research on SSS and suggest that individuals with a more interconnected negative PSS, as well as individuals (and their partners) with a less interconnected positive PSS, report higher daily depressed mood. Unsurprisingly given previous work demonstrating that SSS is a stronger predictor of depressive symptoms than PSS (Murphy et al., 2025; Wilde & Dozois, 2018) the actor effect of PSS on depressed mood was no longer significant when accounting for SSS. However, the partner effect, which remained significant when controlling for SSS, is noteworthy, as, in addition to underscoring the importance of dyadic data, it suggests that the positive beliefs that partner A holds about partner B are associated with lower daily depressed mood for partner B. Though exploratory, assuming these findings replicate, they may have important clinical implications. For example, in conjunction with extant literature linking SSS and depression (Dozois & Beck, 2023), romantic couples and therapists may benefit from an understanding that a variety of beliefs (including a partner’s beliefs about the individual) can all shape an individual’s daily depressed mood.

### Theoretical and applied contributions

Taken together, the present work offers some support for Wilde and Dozois’ (2019) DPSM, specifically its assertion that PSS are key contributors to in-vivo cognitions and behaviors towards romantic partners. For example, the finding that PSS are associated with the cognitions that occur on the day of a conflict (i.e., relationship rumination), lends some support to the notion that PSS impact in-vivo cognitions. Nevertheless, the nonsignificant findings within the conflict severity models suggest that, at least within a non-clinical sample, schema structures are not associated with the perceived severity of negative partner interactions in daily life, but could instead be operating as moderators within this context. Should these findings replicate in more diverse samples (e.g., individuals experiencing depression and/or distressed couples), revisions to the DPSM, clarifying what specific type(s) of cognitions and behaviors may be impacted by schema structures, could be warranted.

This study also offers unique contributions to the broader literature. Extant schema structure research has overwhelmingly focused on the role of SSS in depression (Wilde, 2023; Wilde & Dozois, 2018, 2019). The limited work which has investigated schema structures in depressive symptoms and relational outcomes in non-dyadic, cross-sectional samples has largely found that SSS predict depressive symptoms, whereas PSS are better predictors of relational outcomes (see Murphy et al., 2025). However, the actor and partner effects found within this daily diary sample indicate that these initial findings may be overly simplistic. For example, this is the first study to our knowledge to demonstrate that a partner’s PSS also play a role in shaping actor’s daily depressed mood. This finding, when considered alongside prior research on SSS and depression (see Dozois & Beck, 2023, for a review), may suggest that whereas self-schemas could play a stronger role in predicting depressive symptoms, partner-schemas may still influence daily depressed mood for both dyad members. In line with calls for a more comprehensive focus on the interpersonal context of depression (e.g., Hames et al., 2013; Hammen & Shih, 2014), these results highlight the importance of considering both dyad members’ partner- and self-schema structures in future research on relational- and depressive-related phenomena.

More broadly within the field of relationships research, our findings re-iterate the utility of assessing both positive and negative aspects of couples’ everyday lives, given that we found diverging results depending on the aspect of relationship well-being examined (e.g., relationship quality versus relationship rumination). For instance, negative PSS were predictive of more negative relational aspects (e.g., relationship rumination), but not more positive relationship aspects (e.g., relationship quality).

### Strengths, limitations, and future directions

This study provides the first examination of partner-schemas in daily relationship well-being and depressed mood, and uniquely examines these associations within a dyadic sample. By utilizing this methodology to examine numerous indices of relationship well-being and daily depressed mood in a large sample of couples, this study afforded a strong replication and extension of previous cross-sectional findings (e.g., positive PSS as a significant predictor of relationship satisfaction and commitment; Wilde & Dozois, 2018).

Despite these strengths, several limitations should be noted. Chiefly, the current sample may not be generalizable to a diverse range of couples. The present sample reported high relationship quality, was relatively young, in newer relationships, and highly educated – an unfortunate sampling bias common to many studies within the romantic relationships field (Williamson et al., 2022). Additionally, some socioeconomic status and demographic indictors (e.g., employment status, income, disability information) were not assessed, limiting our understanding of this sample’s demographic characteristics. Although a relative strength of this work was that this large dyadic sample was diverse with respect to ethnicity and sexual orientation, future work should seek to replicate this research among more diverse couples (e.g., older, non-college educated, low-income couples), and clinical samples (e.g., couples experiencing relationship distress or couples in which one partner is experiencing clinical depression).

Beyond concerns of generalizability, PSS were only assessed at baseline. As such, directionality cannot be inferred. Although prior work has established that PSS are stable across time (Wilde, 2023), future research may benefit from examining both short- and long-term longitudinal associations. Indeed, several scholars have underscored the need for — and utility of — multi-timescale studies with intensive longitudinal designs within romantic relationships research (e.g., a daily diary methodology embedded within a longitudinal methodology spanning months and years; Galovan et al., 2023). Finally, it is important to remind readers that less than 15% of all possible diary entries were coded as conflict days, and thus, any models assessing conflict severity were substantially less powered than non-conflict models. Future work that samples couples who experience more frequent conflicts may better elucidate the associations among PSS, relationship quality, relationship rumination, and depressed mood within the context of relational conflict.

## Conclusion

Drawing on relationship distress, depression, and cognitive vulnerability research, the present study sought to explore the role of baseline PSS on daily relationship well-being and depressed mood within a large sample of dyads. The findings suggest that PSS play an important role in shaping various daily well-being indices for oneself and, at times, one’s partner. Moreover, this work underscores the need to replicate effects found in cross-sectional work (e.g., Wilde & Dozois, 2018) within the quotidian lives of couples, and the importance of examining the role of PSS on both relational well-being indices and depressed mood. Supplementary moderation findings also offer preliminary insight into how both couple members’ PSS may shape daily relationship quality and depressed mood when severe conflicts occur in daily life. Future research should attempt to replicate these findings within clinical, and more diverse, samples.

## Supplemental Material

Supplemental Material - A dyadic daily diary investigation of partner-schema structures on relational well-being and depressed mood Supplemental Material for A dyadic daily diary investigation of partner-schema structures on relational well-being and depressed mood by Gabriela C. M. Murphy, Fei Ying, Owen Hicks, Jessica A. Maxwell, David J. A. Dozois in Journal of Social and Personal Relationships

Supplemental Material - A dyadic daily diary investigation of partner-schema structures on relationship well-being and depressed mood Supplemental Material for A dyadic daily diary investigation of partner-schema structures on relationship well-being and depressed mood by Gabriela C. M. Murphy, Fei Ying, Owen Hicks, Jessica A. Maxwell, David J. A. Dozois in Journal of Social and Personal Relationships

## Data Availability

The data and syntax have been made available on the Open Science Framework (OSF) and can be accessed at https://osf.io/prav2.

## Notes

1. This reflects a minor departure from the preregistration, see Supplement for details.

2. This preregistration outlines hypotheses and materials for a large dataset and project, only a portion of which are described within the present manuscript. The preregistration for the larger parent project will be made available once all studies relating to the parent project have been published. For details regarding this preregistration, please contact Gabriela C. M. Murphy. Data for the present study has also been used in the following publications: (Hicks et al., under review; Ying et al., in prep).

3. See Supplement for a priori power analysis details.

4. The preregistration noted that two-level cross-classified models would be used. However, the preregistration also erroneously notes that aggregated daily variables will be used as dependent variables. A two-level cross-classified model is not possible when daily variables are aggregated. To maximize power, we opted to run a two-level cross-classified model with non-aggregated variables.

5. Although relationship rumination was not preregistered as an outcome in these moderation analyses, it was added in light of reviewer suggestions.

## References

[bibr1-02654075251407064] Aguilar-Raab C. Winter F. Jarczok M. N. Ditzen B. Warth M. (2022). Feeling low and unhappy together? An actor-partner-interdependence model uncovering the linkage between different operationalizations of relationship quality and depression in different-sex couples. PLoS One, 17(11), Article e0274756. 10.1371/journal.pone.0274756 36383518 PMC9668111

[bibr2-02654075251407064] Baldwin M. W. (1992). Relational schemas and the processing of social information. Psychological Bulletin, 112(3), 461–484. 10.1037/0033-2909.112.3.461

[bibr3-02654075251407064] Beach S. R. H. Katz J. Kim S. Brody G. H. (2003). Prospective effects of marital satisfaction on depressive symptoms in established marriages: A dyadic model. Journal of Social and Personal Relationships, 20(3), 355–371. 10.1177/0265407503020003005

[bibr4-02654075251407064] Bolger N. Davis A. Rafaeli E. (2003). Diary methods: Capturing life as it is lived. Annual Review of Psychology, 54, 579–616. 10.1146/annurev.psych.54.101601.145030 12499517

[bibr5-02654075251407064] Braithwaite S. Holt-Lunstad J. (2017). Romantic relationships and mental health. Current Opinion in Psychology, 13, 120–125. 10.1016/j.copsyc.2016.04.001 28813281

[bibr6-02654075251407064] Burgoon J. K. (2015). Expectancy violations theory. In Berger C. R. Roloff M. E. Wilson S. R. Dillard J. P. Caughlin J. Solomon D. (Eds.), *The international encyclopedia of interpersonal communication* . Wiley. 10.1002/9781118540190.wbeic102

[bibr7-02654075251407064] Busby D. M. Holman T. B. (2009). Perceived match or mismatch on the Gottman conflict styles: Associations with relationship outcome variables. Family Process, 48(4), 531–545. 10.1111/j.1545-5300.2009.01300.x 19930437

[bibr8-02654075251407064] Campbell L. Simpson J. A. Boldry J. G. Rubin H. (2010). Trust, variability in relationship evaluations, and relationship processes. Journal of Personality and Social Psychology, 99(1), 14–31. 10.1037/a0019714 20565183

[bibr9-02654075251407064] Chatav Y. Whisman M. A. (2009). Partner-schemas and relationship functioning: A states of mind analysis. Behavior Therapy, 40(1), 50–56. 10.1016/j.beth.2007.12.005 19187816

[bibr10-02654075251407064] Cooper A. N. Totenhagen C. J. McDaniel B. T. Curran M. A. (2018). Volatility in daily relationship quality: The roles of attachment and gender. Journal of Social and Personal Relationships, 35(3), 348–371. 10.1177/0265407517690038

[bibr11-02654075251407064] Davila J. Bradbury T. N. Cohan C. L. Tochluk S. (1997). Marital functioning and depressive symptoms: Evidence for a stress generation model. Journal of Personality and Social Psychology, 73(4), 849–861. 10.1037//0022-3514.73.4.849 9325596

[bibr12-02654075251407064] Diehl C. Yin S. Markell H. Gallop R. Gibbons M. B. C. Crits-Christoph P. (2017). The measurement of cognitive schemas: Validation of the psychological distance scaling task in a community mental health sample. International Journal of Cognitive Therapy, 10(1), 17–33. 10.1521/ijct_2016_09_18 29250215 PMC5731789

[bibr13-02654075251407064] Dozois D. J. A. (2002). Cognitive organization of self-schematic content in nondysphoric, mildly dysphoric, and moderately-severely dysphoric individuals. Cognitive Therapy and Research, 26(3), 417–429. 10.1023/A:1016037229820

[bibr14-02654075251407064] Dozois D. J. A. (2007). Stability of negative self-structures: A longitudinal comparison of depressed, remitted, and nonpsychiatric controls. Journal of Clinical Psychology, 63(4), 319–338. 10.1002/jclp.20349 17279521

[bibr15-02654075251407064] Dozois D. J. A. (2021). The importance of social connectedness: From interpersonal schemas in depression to relationship functioning and well-being. Canadian Psychology/Psychologie Canadienne, 62(2), 174–180. 10.1037/cap0000253

[bibr16-02654075251407064] Dozois D. J. A. Beck A. T. (2023). Negative thinking in depression: Cognitive products and schema structures. In Dozois D. J. A. Dobson K. S. (Eds.), Treatment of psychosocial risk factors in depression (pp. 207–232). American Psychological Association.

[bibr17-02654075251407064] Dozois D. J. A. Dobson K. S. (2001a). A longitudinal investigation of information processing and cognitive organization in clinical depression: Stability of schematic interconnectedness. Journal of Consulting and Clinical Psychology, 69(6), 914–925. 10.1037/0022-006X.69.6.914 11777119

[bibr18-02654075251407064] Dozois D. J. A. Dobson K. S. (2001b). Information processing and cognitive organization in unipolar depression: Specificity and comorbidity issues. Journal of Abnormal Psychology, 110(2), 236–246. 10.1037//0021-843x.110.2.236 11358018

[bibr19-02654075251407064] Dozois D. J. A. Frewen P. A. (2006). Specificity of cognitive structure in depression and social phobia: A comparison of interpersonal and achievement content. Journal of Affective Disorders, 90(2–3), 101–109. 10.1016/j.jad.2005.09.008 16343641

[bibr20-02654075251407064] Dozois D. J. A. Wilde J. Dobson K. S. (2020). Depressive disorders. In Antony M. M. Barlow D. H. (Eds.), Handbook of assessment and treatment planning for psychological disorders (3rd ed., pp. 335–378). Guilford Press.

[bibr21-02654075251407064] Eastwick P. W. Finkel E. J. Joel S. (2023). Mate evaluation theory. Psychological Review, 130(1), 211–241. 10.1037/rev0000360 35389716

[bibr22-02654075251407064] Elphinston R. A. Feeney J. A. Noller P. Connor J. P. Fitzgerald J. (2013). Romantic jealousy and relationship satisfaction: The costs of rumination. Western Journal of Communication, 77(3), 293–304. 10.1080/10570314.2013.770161

[bibr23-02654075251407064] Fletcher G. J. O. Simpson J. A. Thomas G. (2000). The measurement of perceived relationship quality components: A confirmatory factor analytic approach. Personality and Social Psychology Bulletin, 26(3), 340–354. 10.1177/0146167200265007

[bibr24-02654075251407064] Fowers B. J. Laurenceau J.-P. Penfield R. D. Cohen L. M. Lang S. F. Owenz M. B. Pasipandoya E. (2016). Enhancing relationship quality measurement: The development of the relationship flourishing scale. Journal of Family Psychology, 30(8), 997–1007. 10.1037/fam0000263 27918187

[bibr25-02654075251407064] Galovan A. M. Orbuch T. L. Shrout M. R. Drebit E. Rice T. M. (2023). Taking stock of the longitudinal study of romantic couple relationships: The last 20 years. Personal Relationships, 30(1), 174–216. 10.1111/pere.12452

[bibr26-02654075251407064] Gilmour A. L. Whisman M. A. Whitton S. W. (2022). A dyadic analysis of relationship satisfaction and depressive symptoms among same-sex couples. Journal of Family Psychology, 36(3), 372–377. 10.1037/fam0000912 34472936

[bibr27-02654075251407064] Gustavson K. Røysamb E. von Soest T. Helland M. J. Karevold E. Mathiesen K. S. (2012). Reciprocal longitudinal associations between depressive symptoms and romantic partners’ synchronized view of relationship quality. Journal of Social and Personal Relationships, 29(6), 776–794. 10.1177/0265407512448264

[bibr28-02654075251407064] Hames J. L. Hagan C. R. Joiner T. E. (2013). Interpersonal processes in depression. Annual Review of Clinical Psychology, 9, 355–377. 10.1146/annurev-clinpsy-050212-185553 23297787

[bibr29-02654075251407064] Hammen C. L. Shih J. (2014). Depression and interpersonal processes. In Gotlib I. H. Hammen C. L. (Eds.), Handbook of depression (3rd ed., pp. 277–295). Guilford Press.

[bibr30-02654075251407064] Hankin B. L. (2008). Stability of cognitive vulnerabilities to depression: A short-term prospective multiwave study. Journal of Abnormal Psychology, 117(2), 324–333. 10.1037/0021-843X.117.2.324 18489208 PMC2756216

[bibr31-02654075251407064] Hicks O. Murphy G. C. M. Ying F. Maxwell J. A. Wilde J. L. Dozois D. J. A. (under review). Core beliefs about you and me: Schema structures, relationship quality, and depressive severity in romantic couples. 10.1016/j.jad.2025.121093 41482269

[bibr32-02654075251407064] Horne R. M. Raposo S. Muise A. Harasymchuk C. Impett E. A. (2022). Dialing up desire and dampening disinterest: Regulating sexual desire in the bedroom and sexual and relationship well-being. Journal of Social and Personal Relationships, 39(6), 1551–1573. 10.1177/02654075211054781 35574184 PMC9092913

[bibr33-02654075251407064] Horwitz A. G. Zhao Z. Sen S. (2023). Peak-end bias in retrospective recall of depressive symptoms on the PHQ-9. Psychological Assessment, 35(4), 378–381. 10.1037/pas0001219 36757996 PMC10052790

[bibr34-02654075251407064] Joel S. Eastwick P. W. Allison C. J. Arriaga X. B. Baker Z. G. Bar-Kalifa E. Wolf S. Birnbaum G. E. Brock R. L. Brumbaugh C. C. Carmichael C. L. Chen S. Clarke J. Cobb R. J. Coolsen M. K. Davis J. de Jong D. C. Debrot A. DeHaas E. C. (2020). Machine learning uncovers the most robust self-report predictors of relationship quality across 43 longitudinal couples studies. Proceedings of the National Academy of Sciences of the United States of America, 117(32), 19061–19071. 10.1073/pnas.1917036117 32719123 PMC7431040

[bibr35-02654075251407064] Kenny D. A. Kashy D. A. Cook W. L. (2006). Dyadic data analysis. Guilford Press.

[bibr36-02654075251407064] Kirkegaard T. (2006). The association between rumination and negative affect: A review. Cognition & Emotion, 20(8), 1216–1235. 10.1080/02699930500473533

[bibr37-02654075251407064] Kluwer E. S. Johnson M. D. (2007). Conflict frequency and relationship quality across the transition to parenthood. Journal of Marriage and Family, 69(5), 1089–1106. 10.1111/j.1741-3737.2007.00434.x

[bibr38-02654075251407064] Laurenceau J.-P. Bolger N. (2005). Using diary methods to study marital and family processes. Journal of Family Psychology, 19(1), 86–97. 10.1037/0893-3200.19.1.86 15796655

[bibr39-02654075251407064] Laursen B. Hafen C. (2010). Future directions in the study of close relationships: Conflict is bad (except when it’s not). Social Development, 19(4), 858–872. 10.1111/j.1467-9507.2009.00546.x 20953335 PMC2953261

[bibr40-02654075251407064] Li X. Cao H. Zhou N. Ju X. Lan J. Zhu Q. Fang X. (2018). Daily communication, conflict resolution, and marital quality in Chinese marriage: A three-wave, cross-lagged analysis. Journal of Family Psychology, 32(6), 733–742. 10.1037/fam0000430 29771550

[bibr41-02654075251407064] Lischetzke T. (2014). Daily diary methodology. In Michalos A. C. (Ed.), Encyclopedia of quality of life and well-being research. Springer. 10.1007/978-94-007-0753-5_657

[bibr42-02654075251407064] Machado D. (2024). Development and validation of a novel partner rumination scale. Western University Open Repository. https://hdl.handle.net/20.500.14721/37722

[bibr43-02654075251407064] Mackinnon S. P. Sherry S. B. Antony M. M. Stewart S. H. Sherry D. L. Hartling N. (2012). Caught in a bad romance: Perfectionism, conflict, and depression in romantic relationships. Journal of Family Psychology, 26(2), 215–225. 10.1037/a0027402 22353007

[bibr44-02654075251407064] Maxwell J. A. Meltzer A. L. (2020). Kiss and makeup? Examining the co-occurrence of conflict and sex. Archives of Sexual Behavior, 49(8), 2883–2892. 10.1007/s10508-020-01779-8 32651881

[bibr45-02654075251407064] Mitchell A. J. (2008). Are one or two simple questions sufficient to detect depression in cancer and palliative care? A Bayesian meta-analysis. British Journal of Cancer, 98(12), 1934–1943. 10.1038/sj.bjc.6604396 18506146 PMC2441968

[bibr46-02654075251407064] Morgan P. Love H. A. Durtschi J. May S. (2018). Dyadic causal sequencing of depressive symptoms and relationship satisfaction in romantic partners across four years. American Journal of Family Therapy, 46(5), 486–504. 10.1080/01926187.2018.1563004

[bibr65-02654075251407064] Murphy G. C. M. Ying F. Wilde J. L. Hicks O. Sivakumar P. Maxwell J. A. Dozois D. J. A. (2025). Assessing the psychometric properties of the partner version of the psychological distance scaling task. Personal Relationships, 32(2). 10.1111/pere.70015

[bibr47-02654075251407064] Pâquet M. Rosen N. O. Steben M. Mayrand M. H. Santerre-Baillargeon M. Bergeron S. (2018). Daily anxiety and depressive symptoms in couples coping with vulvodynia: Associations with women’s pain, women’s sexual function, and both partners’ sexual distress. The Journal of Pain, 19(5), 552–561. 10.1016/j.jpain.2017.12.264 29309891

[bibr48-02654075251407064] Park Y. Impett E. A. MacDonald G. (2021). Generalizability of results from dyadic data: Participation of one versus two members of a romantic couple is associated with breakup likelihood. Personality and Social Psychology Bulletin, 47(2), 232–240. 10.1177/0146167220920167 32458730

[bibr49-02654075251407064] Peeters F. Nicolson N. A. Berkhof J. Delespaul P. deVries M. (2003). Effects of daily events on mood states in major depressive disorder. Journal of Abnormal Psychology, 112(2), 203–211. 10.1037/0021-843x.112.2.203 12784829

[bibr50-02654075251407064] Peetz J. Shimizu J. P. K. Royle C. (2022). Projecting current feelings into the past and future: Better current relationship quality reduces negative retrospective bias and increases positive forecasting bias. Journal of Social and Personal Relationships, 39(8), 2595–2616. 10.1177/02654075221084280

[bibr51-02654075251407064] Schmidt N. B. Schmidt K. L. Young J. E. (1999). Schematic and interpersonal conceptualizations of depression: An integration. In Joiner T. Coyne J. C. (Eds.), The interactional nature of depression (pp. 127–148). American Psychological Association.

[bibr52-02654075251407064] Schneider B. (2014). Interpersonal distress and interpersonal problems associated with depression. Columbia University.

[bibr53-02654075251407064] Seeds P. M. Dozois D. J. (2010). Prospective evaluation of a cognitive vulnerability-stress model for depression: The interaction of schema self-structures and negative life events. Journal of Clinical Psychology, 66(12), 1307–1323. 10.1002/jclp.20723 20715020

[bibr54-02654075251407064] Senkans S. McEwan T. E. Skues J. Ogloff J. R. P. (2016). Development of a relational rumination questionnaire. Personality and Individual Differences, 90, 27–35. 10.1016/j.paid.2015.10.032

[bibr55-02654075251407064] Sheng R. Hu J. Liu X. Xu W. (2022). Longitudinal relationships between insecure attachment and romantic relationship quality and stability in emerging adults: The mediating role of perceived conflict in daily life. Current Psychology, 42(17), 1–11. 10.1007/s12144-021-02668-636468159

[bibr56-02654075251407064] Wade N. G. Vogel D. L. Liao K. Y.-H. Goldman D. B. (2008). Measuring state-specific rumination: Development of the rumination about an interpersonal offense scale. Journal of Counseling Psychology, 55(3), 419–426. 10.1037/0022-0167.55.3.419

[bibr57-02654075251407064] Watkins E. R. Roberts H. (2020). Reflecting on rumination: Consequences, causes, mechanisms and treatment of rumination. Behaviour Research and Therapy, 127, 103573. 10.1016/j.brat.2020.103573 32087393

[bibr58-02654075251407064] Whisman M. A. Delinsky S. S. (2002). Marital satisfaction and an information-processing measure of partner-schemas. Cognitive Therapy and Research, 26(5), 617–627. 10.1023/A:1020305226067

[bibr59-02654075251407064] Whisman M. A. Sbarra D. A. Beach S. R. (2021). Intimate relationships and depression: Searching for causation in the sea of association. Annual Review of Clinical Psychology, 17, 233–258. 10.1146/annurev-clinpsy-081219-103323 33567901

[bibr60-02654075251407064] Wilde J. L. (2023). An empirical examination of the dyadic partner-schema model of relationship distress and depression. Western University Open Repository. https://hdl.handle.net/20.500.14721/37116

[bibr61-02654075251407064] Wilde J. L. Dozois D. J. A. (2018). It’s not me, it’s you: Self- and partner-schemas, depressive symptoms, and relationship quality. Journal of Social and Clinical Psychology, 37(5), 356–380. 10.1521/jscp.2018.37.5.356

[bibr62-02654075251407064] Wilde J. L. Dozois D. J. A. (2019). A dyadic partner-schema model of relationship distress and depression: Conceptual integration of interpersonal theory and cognitive-behavioral models. Clinical Psychology Review, 70, 13–25. 10.1016/j.cpr.2019.03.003 30875565

[bibr63-02654075251407064] Williamson H. C. Bornstein J. X. Cantu V. Ciftci O. Farnish K. A. Schouweiler M. T. (2022). How diverse are the samples used to study intimate relationships? A systematic review. Journal of Social and Personal Relationships, 39(4), 1087–1109. 10.1177/02654075211053849 35655791 PMC9159543

[bibr64-02654075251407064] Ying F. Hicks O. Murphy G. C. M. Maxwell J. A. Dozois D. J. A. (In prep). Beliefs and behaviours: The impact of partner-schema structures on observable interactions between couples.

