# Supplementary material for: A dyadic daily diary investigation of partner-schema structures on relational well-being and depressed mood
Source: J Soc Pers Relat. 2025 Dec 9;43(5):1411–37. doi: 10.1177/02654075251407064 (PMC13044449; doi:10.1177/02654075251407064)
Supplement: Supplemental Material - A dyadic daily diary investigation of partner-schema structures on relational well-being and depressed mood [file sj-pdf-1-spr-10.1177_02654075251407064.pdf]

A Dyadic Daily Diary Investigation of Partner-Schema Structures on Relational Well-Being and Depressed Mood

Supplemental Material

Preregistration Note on Lagged Analyses on Conflict Days

Please note that the hypotheses surrounding lagged analyses on conflict days reflects a minor departure from the preregistration. Specifically, the preregistration originally proposed an examination of relationship quality on the day following a conflict occurrence, after controlling for relationship quality on the conflict day. However, recent literature suggests that relationship quality may be more impacted on the day of a negative relational or stressful experience (Galovan et al., 2023), such as conflict occurrence. In light of this recent evidence, we examined the role relationship quality on the day of a conflict occurrence, rather than the day following a conflict occurrence. Moreover, examining relationship rumination on the day of a conflict occurrence was not preregistered.

Missing Data: Participant Demographics

Three participants were missing demographic data from the baseline survey due to a glitch in the survey software resulting in only some variables being saved. As such, these three participants are not included in the percentages described within the participant section, with the exception of their gender and relationship length.

Missing Data: Schema Structures

Twenty-six individuals across 24 dyads were missing at least one partner-schema structure (PSS) score and were removed prior to data analyses as they would not be included in the main models. Specifically, 26 participants across 24 dyads were missing negative PSS because they did not endorse any negative adjectives as being referent of their partner, and two of these 26 participants were also missing positive PSS because they did not endorse any positive adjectives as being referent of their partner.

Of these 26 cases of missing schema structures, two cases were considered missing at

random: two participants did not have a positive nor negative PSS score as a result of the previously described glitch in the baseline survey (these participants were also missing demographic information). The remaining 24 cases are considered missing not at random.

Because the vast majority of these cases were not considered missing at random, we tested whether individuals missing a PSS score varied from those who were not missing PSS scores on all key dependent, control, and demographic variables. Results of independent samples t-tests comparing the 26 individuals who were removed for missing one or both partner-schema structure scores against the 542 individuals with both positive and negative partner schema structure scores suggested that these two groups did not differ significantly on aggregated scores of daily relationship quality, relationship rumination, conflict severity, nor depressed mood. With regard to demographic variables, participants did not significantly differ in age nor years in education. There were insufficient cell counts for each value within the categorical variables of gender, relationship status, and sexual orientation for Chi-Squared comparisons. Of the 26 individuals who did not have a partner schema structure score, 19 were seriously dating one person and seven were married or common-law. With respect to gender, 11 individuals were cisgender men, and 15 were cisgender women. Finally, with respect to sexual orientation, 21 identified as heterosexual, one as asexual, one as bisexual, and one as lesbian.

Psychological Distance Scaling Task (PDST) Instructions and Word Lists

Distance Scaling Task (Partner) - Instructions

Please read carefully.

On the next screen you will see a graph. On the extreme right is the description "Very much like my partner" and on the left is the description "Not at all like my partner". The other line refers to how positive or negative you think each word is. At the top is the description "Positive" and at the bottom is the description "Negative".

In the center of the graph, a word will appear. Your task is to determine both **how much or how little the word describes YOUR PARTNER** AND **how positive or negative the word is**. You must choose a location on the graph and click there to indicate where you think the word fits. A dot will appear where you click the graph. You may click the mouse anywhere in the grid. The more the word describes your partner, the further to the right you should place it, and the less the word describes your partner, the further to the left you should place it. The more positive you believe the word to be, the higher on the graph you should place it, and the more negative you believe the word to be, the lower on the graph you should place it. In the example below, the person believed that the word "PLAYFUL" describes his/her partner moderately well and thought that this word is very positive (note the location of the dot).

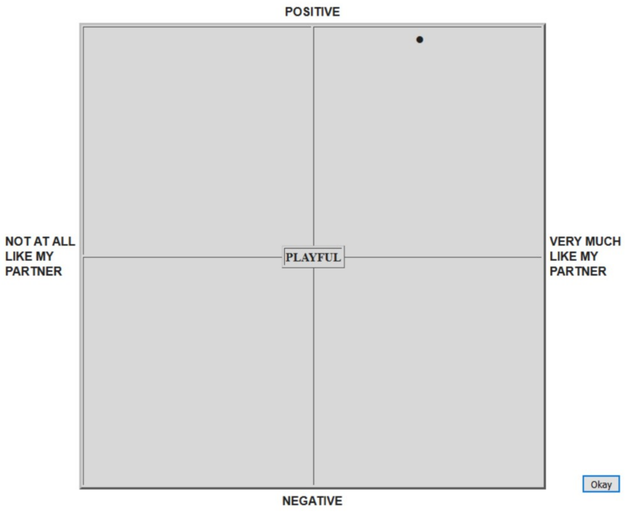

After you have made your selection and are satisfied with your response, click 'Okay' or press the 'Enter' key on the keyboard, and the next word will appear. If you are not satisfied with your response, just move the mouse to the position you wish and click the mouse button again. Remember, you are actually making 2 decisions here: one for how much each word describes **YOUR PARTNER** AND one for how positive or negative you think the word is. **Please continue until you have completed all the words**. This task will take about 5 minutes to complete.

The first four words are practice trials, so you can get used to the task. If you have any questions or are unsure about how to do the task, please ask the experimenter for help.

Distance Scaling Task (Self) - Instructions

Please read carefully.

On the next screen you will see a graph. On the extreme right is the description "Very much like me" and on the left is the description "Not at all like me". The other line refers to how positive or negative you think each word is. At the top is the description "Positive" and at the bottom is the description "Negative".

In the center of the graph, a word will appear. Your task is to determine both **how much or how little the word describes YOU** AND **how positive or negative the word is**. You must choose a location on the graph and click there to indicate where you think the word fits. A dot will appear where you click the graph. You may click the mouse anywhere in the grid. The more the word describes you, the further to the right you should place it, and the less the word describes you, the further to the left you should place it. The more positive you believe the word to be, the higher on the graph you should place it, and the more negative you believe the word to be, the lower on the graph you should place it. In the example below, the person believed that the word "KIND" describes himself/herself moderately well and thought that this word is very positive (note the location of the dot).

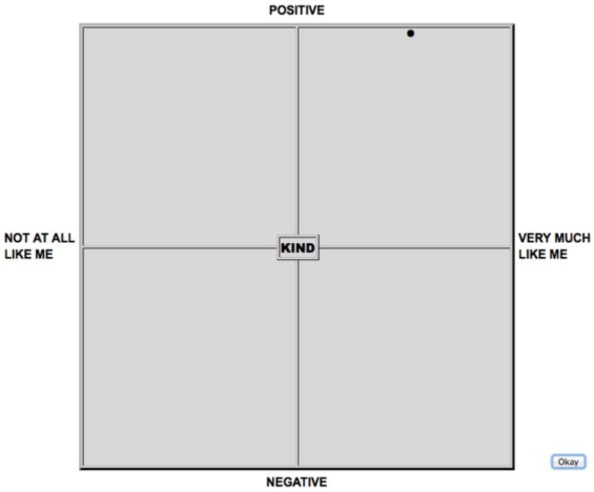

After you have made your selection and are satisfied with your response, click 'Okay' or press the 'Enter' key on the keyboard, and the next word will appear. If you are not satisfied with your response, just move the mouse to the position you wish and click the mouse button again. Remember, you are actually making 2 decisions here: one for how much each word describes **YOU** AND one for how positive or negative you think the word is. **Please continue until you have completed all the words**. This task will take about 5 minutes to complete.

The first four words are practice trials, so you can get used to the task. If you have any questions or are unsure about how to do the task, please ask the experimenter for help.

Continue to Task

Adjective Lists for the PDST

**Positive**	**Negative**
1. Admired	31. Aggressive
2. Approving	32. Alone
3. Comical	33. Annoying
4. Communicative	34. Attention-seeker
5. Confiding	35. Bossy
6. Connected	36. Combative
7. Delightful	37. Controlling
8. Dependable	38. Criticizing
9. Encouraging	39. Demanding
10. Energetic	40. Dependent
11. Entertaining	41. Forceful
12. Extroverted	42. Gossiper
13. Gentle	43. Hot-tempered
14. Gifted	44. Immature
15. Gracious	45. Impatient
16. Hilarious	46. Insecure
17. Humble	47. Irritable
18. Joyful	48. Judgmental
19. Lively	49. Lazy
20. Marvelous	50. Lonely
21. Neighbourly	51. Lonesome
22. Nonjudgmental	52. Needy
23. Outgoing	53. Overbearing
24. Playful	54. Pessimistic
25. Respected	55. Possessive
26. Selfless	56. Pushy
27. Soft-hearted	57. Quarrelsome
28. Spontaneous	58. Resentful
29. Valuable	59. Showy
30. Wonderful	60. Unassertive

During the development of the PDST, word lists were balanced with respect to word length, frequency of word usage, and degree of emotionality (Dozois & Dobson, 2001).

Power Analysis

A detailed description of the a priori power analyses for all main analyses preregistered with this data (i.e., including non-daily, cross-sectional analyses) can be found within this study's preregistration on the Open Science Framework. Specifically, we note: "In accordance with the limits of our funding allocation, we will be collecting a total of 300 dyads before conducting our main analyses. To ascertain the magnitude of effects we can detect with this sample size, we conducted two a priori power analyses using the "APIMPower: An interactive tool for Actor-Partner Interdependence Model power analysis" web app (Ackerman & Kenny, 2016). We focused on our power to detect partner effects, as these are typically smaller in magnitude than actor effects. Results suggest that a sample size of 167 dyads is needed to detect a partner effect of $r = .15$ (when the actor effect, correlation between partners, and correlation of errors are set to $r = .2$), with 80% power. Or, if we suppose that our 300 dyads will result in 80% complete data (a sample size of 240 dyads), we would have 82% power to detect a small partner effect of $r = .13$ when all other estimates (actor effect, correlation between partners and correlation of errors) are conservatively set to $r = .13$. In other words, our sample size of 300 dyads will allow us to detect effects of a small magnitude." (p. 24, Hicks et al., 2022). Based on these a priori estimates, and with statistical power maximized by collecting repeated (e.g., daily) assessments of key outcomes (Sisson et al., 2022), the present sample was estimated to provide sufficient power to detect small effects.

Effect Size Calculations

All effect sizes (*r*) were calculated using the formula: $r = \sqrt{(t2 / t2 + df)}$ (Rosnow & Rosenthal, 2007), a common approach for calculating effect sizes in multi-level models, particularly with dyadic and daily diary samples (e.g., Harrington et al., 2022).

Table S1

Bivariate Correlations Among Study Variables

	ICC	1	2	3	4	5	6	7
1. PSS+	—	**.08**†						
2. PSS-	—	-.15***	**.04**					
3. Rel. Quality	0.61	-.23***	.06	**.66*** **				
4. Conflict Severity	0.31	.06	-.12*	-.29***	**.44*** **			
5. Rel. Rumination	0.42	.17***	-.21***	-.38***	.58***	**.42*** **		
6. Depressed Mood	0.39	.17***	-.15***	-.27***	.35***	.57***	**.19*** **	
7. Rel. Length	—	.08†	.01	-.04	-.15**	-.14**	-.10*	—

Note. Correlations below the diagonal are between the actor variables. When calculating bivariate correlations, daily variables were aggregated within-person across all entries of the 14-day diary period, a common approach to examine bivariate correlations within dyadic daily diary data (e.g., Horne et al., 2022; Vaillancourt-Morel et al., 2020). Bolded correlations along the diagonal are between the actor and partner variables. PSS+ = positive partner-schema structure interstimulus distance; PSS- = negative partner-schema structure interstimulus distance; Rel. = Relationship*;* ICC = Intraclass Correlation.

**** p <.001; ** p < .01; * p < .05; †p <.10.*

Partner Schema Structures Predicting Conflict Severity

Table S2

PSS Predicting Actors' Daily Conflict Severity

	Actor Daily Conflict Severity					
**Effects**	***b***	***SE***	***t(df)***	***p***	**95% CI**	**Effect size *r***
Actor						
PSS+	0.33	0.37	0.88 (405.13)	.382	[-0.41, 1.06]	.04
PSS-	-0.36	0.19	-1.93 (350.31)	.054	[-0.73, 0.01]	.10
Partner						
PSS+	0.14	0.35	0.41 (375.76)	.685	[-0.54, 0.82]	.02
PSS-	-0.27	0.18	-1.48 (338.79)	.141	[-0.63, 0.09]	.08

Note. PSS+ = positive partner-schema structure interstimulus distance; PSS- = negative partner-schema structure interstimulus distance.

Generalizability of Findings

Relationship Length

Previous work suggests that relationship well-being and depressive symptoms may be differentially impacted by the duration of one's relationship (Proulx & Buehler, 2007; Turner & McNulty, 2020; Whitton & Kuryluk, 2012). Although, as shown in Table S1, only positive self-schema structures were marginally significantly associated with relationship length, considering the novelty of this work, as well as the significant correlations between relationship length and many of the outcome variables, relationship length was included in all models as a covariate. Within these models, all significant effects remained, with the exception of actor and partner negative PSS predicting relationship rumination on the day of a conflict, which became marginally significant ($p < .10$). Tables S3-S9 present all main models when controlling for relationship length.

Daily Relationship Quality

Table S3

Partner-Schema Structures Predicting Actor Daily Relationship Quality while Controlling for Relationship Length

	Actor Daily Relationship Quality					
**Effects**	***b***	***SE***	***t(df)***	***p***	**95% CI**	**Effect size *r***
Actor						
**PSS+**	**-0.95**	**0.19**	**-5.10 (267.44)**	**<.001**	**[-1.32, -0.58]**	**.30**
PSS-	0.05	0.10	0.47 (266.67)	.639	[-0.15, 0.24]	.03
Partner						
PSS+	-0.28	0.19	-1.52 (268.53)	.130	[-0.65, 0.08]	.09
PSS-	0.07	0.10	0.67 (266.6)	.503	[-0.13, 0.26]	.04
Rel. Length	-0.01	0.01	-1.92 (316.16)	.056	[-0.03, 0.00]	.11

Note. PSS+ = positive partner-schema structure interstimulus distance; PSS- = negative partner-schema structure interstimulus distance. Rel. = Relationship. Significant effects are in boldface.

Daily Relationship Rumination

Table S4

Partner-Schema Structures Predicting Actor Daily Relationship Rumination while Controlling for Relationship Length

	**Actor Daily Relationship Rumination**					
**Effects**	***b***	***SE***	***t(df)***	***p***	**95% CI**	**Effect size *r***
Actor						
**PSS+**	**0.65**	**0.19**	**3.49 (298.56)**	**<.001**	**[0.29, 1.02]**	**.20**
**PSS-**	**-0.46**	**0.10**	**-4.69 (297.44)**	**<.001**	**[-0.66, -0.27]**	**.26**
Partner						
PSS+	0.21	0.19	1.12 (301.39)	.265	[-0.16, 0.58]	.06
PSS-	-0.15	0.10	-1.54 (297.14)	.126	[-0.35, 0.04]	.09
**Rel. Length**	**-0.02**	**0.01**	**-2.64 (269.45)**	**.009**	**[-0.03, -0.01]**	**.16**

Note. PSS+ = positive partner-schema structure interstimulus distance; PSS- = negative partner-schema structure; Rel. = relationship.

Relationship Quality and Relationship Rumination on the Day of a Conflict Occurrence

Table S5

Partner-Schema Structures Predicting Actors' Daily Relationship Quality While Controlling for Relationship Rumination the Day Prior and Relationship Length

	Actor Daily Relationship Quality on the Day of a Conflict					
**Effects**	***b***	***SE***	***t(df)***	***p***	**95% CI**	**Effect size *r***
Actor						
PSS+	-0.24	0.2	-1.2 (244.56)	.231	[-0.63, 0.15]	.08
PSS-	0.11	0.11	1 (210.17)	.316	[-0.1, 0.32]	.07
Partner						
PSS+	-0.16	0.2	-0.77 (256.27)	.441	[-0.56, 0.24]	.05
PSS-	0.06	0.11	0.56 (211.97)	.577	[-0.15, 0.27]	.04
**Actor Lagged RQ**	**0.64**	**0.03**	**18.73 (587.96)**	**<.001**	**[0.57, 0.71]**	**.61**
Rel. Length	0.01	0.01	1.04 (117.8)	.303	[-0.01, 0.02]	.10

Note. PSS+ = positive partner-schema structure interstimulus distance; PSS- = negative partner-schema structure interstimulus distance. Actor Lagged RQ = actor's relationship quality the day prior; Rel. = Relationship. Significant effects are in boldface

Table S6

Partner-Schema Structures Predicting Actors' Daily Relationship Rumination While Controlling for Relationship Rumination the Day Prior and Relationship Length

	Daily Relationship Rumination on the Day of a Conflict					
**Effects**	***b***	***SE***	***t(df)***	***p***	**95% CI**	**Effect size *r***
Actor						
**PSS+**	**0.75**	**0.35**	**2.11 (288.43)**	**.036**	**[0.05, 1.45]**	**.12**
PSS-	-0.34	0.19	-1.77 (264.55)	.078	[-0.72, 0.04]	.11
Partner						
PSS+	0.23	0.37	0.62 (311.74)	.538	[-0.5, 0.95]	.04
PSS-	-0.37	0.19	-1.89 (265.07)	.059	[-0.74, 0.01]	.12
**Actor Lagged RR**	**0.39**	**0.04**	**10.18 (795.36)**	**<.001**	**[0.31, 0.47]**	**.34**
**Rel. Length**	**-0.03**	**0.01**	**-2.97 (140.05)**	**.004**	**[-0.06, -0.01]**	**.24**

Note. PSS+ = positive partner-schema structure interstimulus distance; PSS- = negative partner-schema structure interstimulus distance. Actor Lagged RR = actor's relationship rumination the day prior; Rel. = Relationship. Significant effects are in boldface.

Daily Conflict Severity

Table S7

Partner-Schema Structures Predicting Actors' Daily Conflict Severity While Controlling Relationship Length

	Actor Daily Conflict Severity					
**Effects**	***b***	***SE***	***t(df)***	***p***	**95% CI**	**Effect size *r***
Actor						
PSS+	0.43	0.37	1.16 (406.77)	.248	[-0.3, 1.16]	.06
PSS-	-0.32	0.19	-1.73 (350.71)	.084	[-0.69, 0.04]	.09
Partner						
PSS+	0.20	0.34	0.58 (375.42)	.564	[-0.48, 0.87]	.03
PSS-	-0.22	0.18	-1.23 (342.14)	.219	[-0.58, 0.13]	.07
**Rel. Length**	**-0.03**	**0.01**	**-2.54 (215.73)**	**.012**	**[-0.05, -0.01]**	**.17**

Note. PSS+ = positive partner-schema structure interstimulus distance; PSS- = negative partner-schema structure interstimulus distance. Rel. = Relationship. Significant effects are in boldface.

Table S8

Partner-Schema Structures Predicting Actors' Daily Depressed Mood While Controlling Relationship Length

	Actor Daily Depressed Mood					
**Effects**	***b***	***SE***	***t(df)***	***p***	**95% CI**	**Effect size**
Actor						
**PSS+**	**0.85**	**0.20**	**4.25 (324.58)**	**<.001**	**[0.46, 1.24]**	**.23**
**PSS-**	**-0.33**	**0.11**	**-3.07 (322.39)**	**.002**	**[-0.53, -0.12]**	**.17**
Partner						
**PSS+**	**0.61**	**0.20**	**3.04 (328.34)**	**.003**	**[0.22, 1.01]**	**.17**
PSS-	0.00	0.11	0.02 (321.70)	.984	[-0.21, 0.21]	.00
**Rel. Length**	**-0.02**	**0.01**	**-2.61 (261.04)**	**.009**	**[-0.03, -0.01]**	**.16**

Note. PSS+ = positive partner-schema structure interstimulus distance; PSS- = negative partner-schema structure interstimulus distance. Rel. = Relationship. Significant effects are in boldface.

Controlling for Self-Schema Structures (SSS)

Previous work suggests that self- and partner-schemas are distinct constructs [Murphy et al., 2025]. Nevertheless, we sought to assess whether significant effects of PSS remained when controlling for SSS. Self-schema structures were assessed using the PDST self-version, which utilizes the same task, adjectives, instructions, and formula as the PDST partner-version. However, rather than the X axis reading "*Not at all like my partner*" on the left, and "*Very much like my partner*", the x-axis reads, "*Not at all like me*" on the left, and "*Very much like me*" on the right (see also pages 4-6 of this Supplement). Fourteen individuals were missing a negative SSS, and two of these 14 individuals were also missing a self-schema structure. Consequently, these 14 individuals and their partners were not included in this model (n = 246 dyads). The SSS scores of the remining 492 individuals were similarly logarithmically transformed with a base 10 (positive self-schema structure score (SSS; M = 0.99, SD = 0.19), and a negative SSS score (M = 1.40, SD =0.27). See Tables S9-S14.

Table S9

Partner-Schema Structures Predicting Actors' Relationship Quality While Controlling for Self-Schema Structures

	Actor Daily Relationship Quality					
**Effects**	***b***	***SE***	***t(df)***	***p***	**95% CI**	**Effect size *r***
Actor						
**PSS+**	**-0.92**	**0.23**	**-3.92 (253.28)**	**<.001**	**[-1.38, -0.46]**	**0.24**
PSS-	-0.01	0.12	-0.06 (252.96)	0.953	[-0.24, 0.23]	0.00
SSS+	-0.18	0.22	-0.84 (256.61)	0.399	[-0.61, 0.24]	0.05
SSS-	0.20	0.14	1.46 (255.51)	0.145	[-0.07, 0.46]	0.09
Partner						
PSS+	-0.34	0.23	-1.44 (254.28)	0.151	[-0.8, 0.12]	0.09
PSS-	0.04	0.12	0.3 (252.35)	0.763	[-0.2, 0.27]	0.02
SSS+	0.01	0.22	0.06 (256.23)	0.952	[-0.41, 0.44]	0.00
SSS-	0.02	0.14	0.17 (255.03)	0.862	[-0.24, 0.29]	0.01

Note. PSS+ = positive partner-schema structure interstimulus distance; PSS- = negative partner-schema structure interstimulus distance; SSS+ = positive self-schema structure interstimulus distance; SSS- = negative Self-schema structure interstimulus distance. Significant effects are in boldface.

Table S10

Partner-Schema Structures Predicting Actors' Relationship Rumination While Controlling for Self-Schema Structures

	Actor Daily Relationship Rumination					
**Effects**	***b***	***SE***	***t(df)***	***p***	**95% CI**	**Effect size *r***
Actor						
**PSS+**	**0.82**	**0.24**	**3.46 (272.48)**	**<.001**	**[0.35, 1.29]**	**0.21**
**PSS-**	**-0.42**	**0.12**	**-3.50 (271.05)**	**<.001**	**[-0.65, -0.18]**	**0.21**
SSS+	-0.24	0.22	-1.1 (282.04)	0.273	[-0.68, 0.19]	0.07
SSS-	-0.16	0.14	-1.18 (279.39)	0.237	[-0.43, 0.11]	0.07
Partner						
PSS+	0.43	0.24	1.82 (275.09)	0.071	[-0.04, 0.90]	0.11
PSS-	-0.07	0.12	-0.62 (269.52)	0.538	[-0.31, 0.16]	0.04
**SSS+**	**-0.47**	**0.22**	**-2.15 (281.09)**	**0.032**	**[-0.91, -0.04]**	**0.13**
**SSS-**	**-0.33**	**0.14**	**-2.39 (278.1)**	**0.017**	**[-0.6, -0.06]**	**0.14**

Note. PSS+ = positive partner-schema structure interstimulus distance; PSS- = negative partner-schema structure interstimulus distance; SSS+ = positive self-schema structure interstimulus distance; SSS- = negative Self-schema structure interstimulus distance. Significant effects are in boldface.

Table S11

Partner-Schema Structures Predicting Actors' Relationship Quality on the day of a conflict While Controlling for Self-Schema Structures

	Actor Daily Relationship Quality on the day of a conflict					
**Effects**	***b***	***SE***	***t(df)***	***p***	**95% CI**	**Effect size *r***
Actor						
PSS+	-0.26	0.25	-1.06 (214.12)	0.291	[-0.75, 0.23]	0.07
PSS-	0.14	0.13	1.03 (190.73)	0.303	[-0.12, 0.39]	0.07
SSS+	-0.08	0.25	-0.34 (258.05)	0.737	[-0.58, 0.41]	0.02
SSS-	0.05	0.16	0.3 (224.07)	0.761	[-0.27, 0.37]	0.02
Partner						
PSS+	-0.26	0.25	-1.04 (230.38)	0.301	[-0.77, 0.24]	0.07
PSS-	0.07	0.13	0.56 (191.43)	0.573	[-0.18, 0.33]	0.04
SSS+	0.19	0.25	0.75 (254.53)	0.454	[-0.3, 0.67]	0.05
SSS-	-0.08	0.16	-0.49 (220.18)	0.622	[-0.4, 0.24]	0.03
**Actor lagged RQ**	**0.62**	**0.04**	**16.74 (541.34)**	**<.001**	**[0.54, 0.69]**	**0.58**

Note. PSS+ = positive partner-schema structure interstimulus distance; PSS- = negative partner-schema structure interstimulus distance; SSS+ = positive self-schema structure interstimulus distance; SSS- = negative Self-schema structure interstimulus distance. Significant effects are in boldface.

Table S12

Partner-Schema Structures Predicting Actors' Relationship Rumination on the day of a conflict While Controlling for Self-Schema Structures

	Actor Daily Relationship Rumination on the Day of a Conflict					
**Effects**	***b***	***SE***	***t(df)***	***p***	**95% CI**	**Effect size *r***
Actor						
**PSS+**	**1.06**	**0.44**	**2.41 (248.93)**	**0.017**	**[0.19, 1.92]**	**0.15**
PSS-	-0.44	0.23	-1.89 (233.95)	0.060	[-0.9, 0.02]	0.12
SSS+	-0.59	0.44	-1.34 (294.36)	0.181	[-1.46, 0.28]	0.08
SSS-	0.05	0.29	0.16 (265.49)	0.875	[-0.53, 0.62]	0.01
Partner						
PSS+	0.39	0.45	0.87 (272.19)	0.387	[-0.5, 1.28]	0.05
**PSS-**	**-0.50**	**0.23**	**-2.15 (231.91)**	**0.032**	**[-0.97, -0.04]**	**0.14**
SSS+	-0.46	0.44	-1.05 (288.45)	0.296	[-1.32, 0.4]	0.06
SSS-	0.25	0.29	0.85 (259.34)	0.394	[-0.32, 0.82]	0.05
**Actor lagged RR**	**0.39**	**0.04**	**9.9 (728.03)**	**<.001**	**[0.31, 0.47]**	**0.34**

Note. PSS+ = positive partner-schema structure interstimulus distance; PSS- = negative partner-schema structure interstimulus distance; SSS+ = positive self-schema structure interstimulus distance; SSS- = negative self-schema structure interstimulus distance. Significant effects are in boldface.

Table S13

Partner-Schema Structures Predicting Actors' Daily Depressed Mood While Controlling for Self-Schema Structures

	Actor Daily Depressed Mood					
**Effects**	***b***	***SE***	***t(df)***	***p***	**95% CI**	**Effect size *r***
Actor						
PSS+	0.36	0.25	1.44 (291.76)	.151	[-0.13, 0.84]	0.08
PSS-	-0.23	0.12	-1.82 (289.11)	.070	[-0.47, 0.02]	0.11
**SSS+**	**0.83**	**0.23**	**3.58 (307.21)**	**<.001**	**[0.37, 1.28]**	**0.20**
**SSS-**	**-0.39**	**0.14**	**-2.69 (302.86)**	**.008**	**[-0.67, -0.1]**	**0.15**
Partner						
**PSS+**	**0.66**	**0.25**	**2.67 (295.43)**	**.008**	**[0.17, 1.15]**	**0.15**
PSS-	-0.02	0.12	-0.15 (286.71)	.884	[-0.26, 0.23]	0.01
SSS+	-0.29	0.23	-1.24 (305.15)	.218	[-0.74, 0.17]	0.07
SSS-	-0.17	0.14	-1.17 (301.02)	.244	[-0.45, 0.12]	0.07

Note. PSS+ = positive partner-schema structure interstimulus distance; PSS- = negative partner-schema structure interstimulus distance; SSS+ = positive self-schema structure interstimulus distance; SSS- = negative Self-schema structure interstimulus distance. Significant effects are in boldface.

Table S14

Partner-Schema Structures Predicting Actors' Conflict Severity While Controlling for Self-Schema Structures

	Actor Daily Conflict Severity					
**Effects**	***b***	***SE***	***t(df)***	***p***	**95% CI**	**Effect size *r***
Actor						
PSS+	0.87	0.45	1.91 (348)	0.057	[-0.02, 1.76]	0.10
**PSS-**	**-0.58**	**0.22**	**-2.62 (321.52)**	**0.009**	**[-1.02, -0.14]**	**0.14**
SSS+	-0.54	0.42	-1.29 (364.15)	0.198	[-1.38, 0.29]	0.07
SSS-	0.11	0.28	0.4 (382.25)	0.691	[-0.44, 0.67]	0.02
Partner						
PSS+	0.37	0.41	0.89 (310.22)	0.375	[-0.45, 1.18]	0.05
PSS-	-0.34	0.21	-1.58 (292.23)	0.115	[-0.76, 0.08]	0.09
SSS+	-0.67	0.44	-1.54 (386.61)	0.125	[-1.54, 0.19]	0.08
SSS-	0.25	0.27	0.95 (344.19)	0.343	[-0.27, 0.78]	0.05

Note. PSS+ = positive partner-schema structure interstimulus distance; PSS- = negative partner-schema structure interstimulus distance; SSS+ = positive self-schema structure interstimulus distance; SSS- = negative Self-schema structure interstimulus distance. Significant effects are in boldface.

Figure S1

Supplementary Moderation Analyses of Partner Schema Structures Predicting Conflict Severity at the Within-Person Level of Conflict Severity

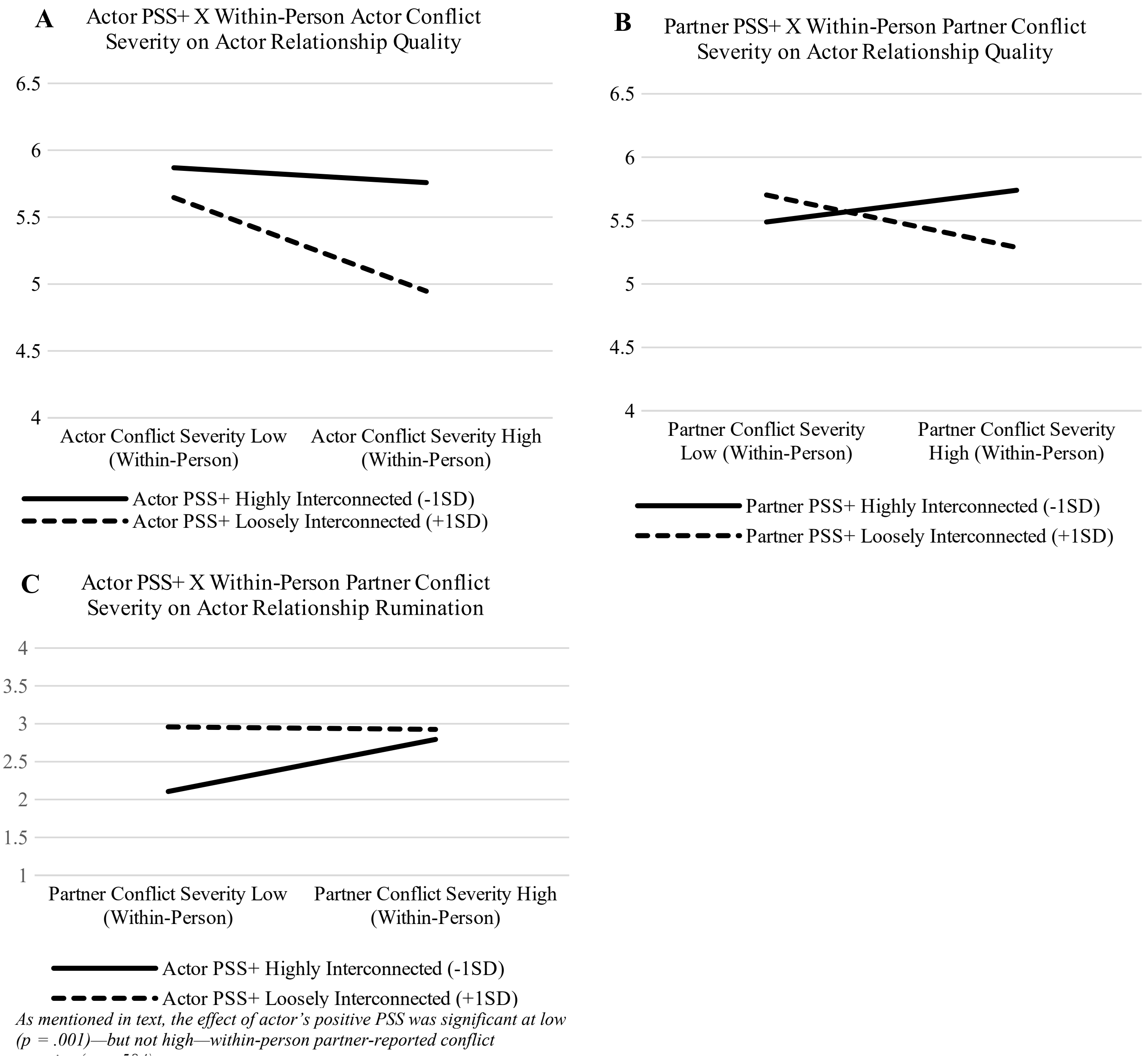

Note. The solid line represented highly interconnected positive partner schema structures (PSS) at -1 *SD* of Interstimulus Distance, and the dashed line represents loosely interconnected positive partner schema structures (PSS) at -1 *SD* of Interstimulus Distance.

Figure S2

Supplementary Moderation Analyses of Partner Schema Structures Predicting Conflict Severity at the Between-Person Level of Conflict Severity

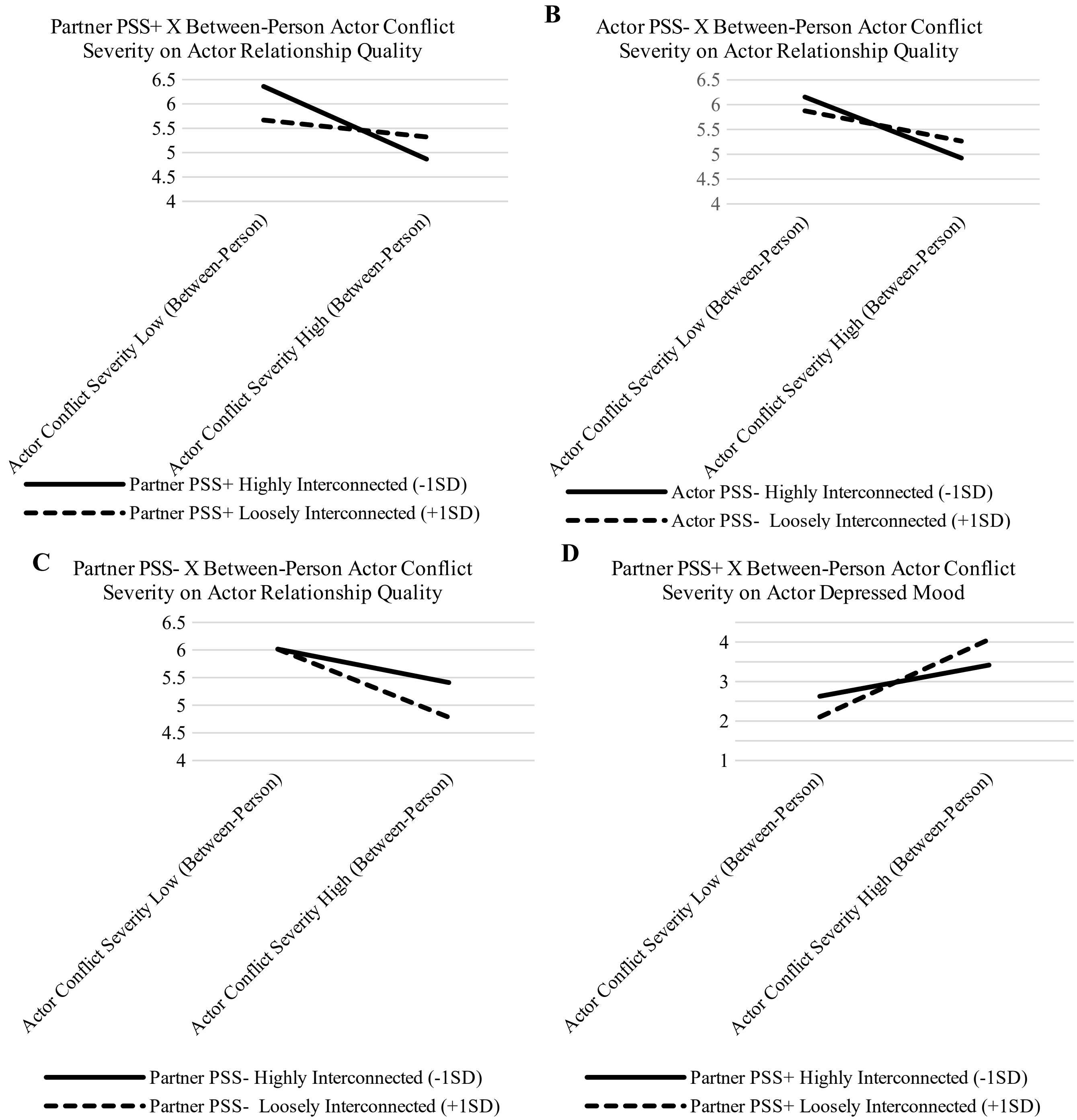

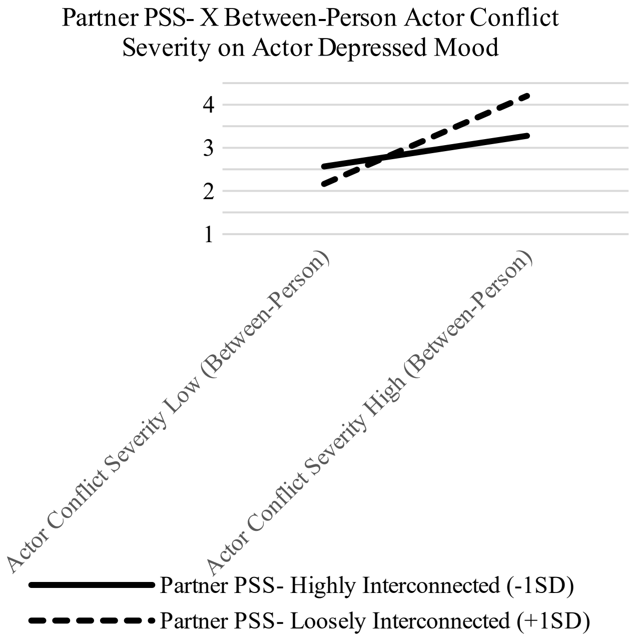

Note. The solid line represented highly interconnected partner schema structures (PSS) at -1 *SD* of Interstimulus Distance, and the dashed line represents loosely interconnected partner schema structures (PSS) at -1 *SD* of Interstimulus Distance.

References

Ackerman, R. A., & Kenny, D. A. (2016, December). APIMPower: An interactive tool for Actor-Partner Interdependence Model power analysis [Computer software]. Available from https://robert-a-ackerman.shinyapps.io/apimpower/

Galovan, A. M., Orbuch, T. L., Shrout, M. R., Drebit, E., & Rice, T. M. (2023). Taking stock of the longitudinal study of romantic couple relationships: The last 20 years. *Personal Relationships*, 30(1), 174–216. https://doi.org/10.1111/pere.12452

Harrington, A. G., Overall, N. C., & Maxwell, J. A. (2022). Feminine gender role discrepancy strain and women's self-esteem in daily and weekly life: A person x context perspective. *Sex Roles*, *87*(1-2), 35–51. https://doi.org/10.1007/s11199-022-01305-1

Hicks, O., Dozois, D. J. A., Murphy, G., Ying, F., & Rain, S. (2022, July 23). STAR (Studying Thoughts and Actions in Relationships). Retrieved from https://osf.io/axheg/?view_only=4f58f90d2a004e9a8d5a88ca295cfbf7

Horne, R. M., Raposo, S., Muise, A., Harasymchuk, C., & Impett, E. A. (2022). Dialing up desire and dampening disinterest: Regulating sexual desire in the bedroom and sexual and relationship well-being. *Journal of Social and Personal Relationships*, *39*(6), 1551–1573. https://doi.org/10.1177/02654075211054781

Dozois, D. J. A., & Dobson, K. S. (2001). Information processing and cognitive organization in unipolar depression: Specificity and comorbidity issues. *Journal of Abnormal psychology*, *110*(2), 236–246. https://doi.org/10.1037//0021-843x.110.2.236

Murphy, G. C. M., Ying, F., Wilde, J. L., Hicks, O., Sivakumar, P., Maxwell, J. A., & Dozois, D. J. A. (2025). Assessing the psychometric properties of the partner version of the psychological distance scaling task. *Personal Relationships*, *32*(2), 1–19.

https://doi.org/10.1111/pere.70015

Proulx, H. M., & Buehler, C. (2007). Marital quality and personal well-being: A meta-analysis. *Journal of Marriage and Family*, *69*(3), 576–593. https://doi.org/10.1111/j.1741-3737.2007.00393.x

Rosnow, R. L., & Rosenthal, R. (2007). Assessing the effect size of outcome research. In A. M. Nezu, and C. M. Nezu (Eds.), *Evidence-based outcome research: a practical guide to conducting randomized controlled trials for psychosocial interventions* (pp. 379-402). University Press. https://doi.org/10.1093/med:psych/9780195304633.003.0018

Sisson, N. M., Wang, G. A., Le, B. M., Stellar, J. E., & Impett, E. A. (2022). When we're asked to change: The role of suppression and reappraisal in partner change outcomes. *Journal of Social and Personal Relationships*, *39*(8), 2388–2407. https://doi.org/10.1177/02654075221078881

Turner, J. A., & McNulty, J. K. (2020). Automatic partner attitudes and daily experiences of mood and relationship satisfaction. *Journal of Family Psychology, 34*(8), 1036–1045. https://doi.org/10.1037/fam0000665

Vaillancourt-Morel, M.-P., Rosen, N. O., Willoughby, B. J., Leonhardt, N. D., & Bergeron, S. (2020). Pornography use and romantic relationships: A dyadic daily diary study. *Journal of Social and Personal Relationships*, *37*(10–11), 2802–2821. https://doi.org/10.1177/0265407520940048

Whitton, S. W., & Kuryluk, A. D. (2012). Relationship satisfaction and depressive symptoms in emerging adults: Cross-sectional associations and moderating effects of relationship characteristics. *Journal of Family Psychology, 26*(2), 226–235. https://doi.org/10.1037/a0027267
